# Prevalence of Sarcoidosis-Associated Pulmonary Hypertension: A Systematic Review and Meta-Analysis

**DOI:** 10.3389/fcvm.2021.809594

**Published:** 2022-01-17

**Authors:** Shijie Zhang, Xiang Tong, Tianli Zhang, Dongguang Wang, Sitong Liu, Lian Wang, Hong Fan

**Affiliations:** Department of Respiratory and Critical Care Medicine, West China Hospital/West China School of Medicine, Sichuan University, Chengdu, China

**Keywords:** sarcoidosis-associated pulmonary hypertension, sarcoidosis, pulmonary hypertension, prevalence, meta-analysis

## Abstract

**Background:**

Sarcoidosis-associated pulmonary hypertension (SAPH) is associated with poor prognosis, conferring up to a 10-fold increase in mortality in patients with sarcoidosis, but the actual prevalence of SAPH is unknown.

**Methods:**

The PubMed, Embase, and Cochrane Library databases were systematically searched for epidemiological studies reporting the prevalence of SAPH up to July 2021. Two reviewers independently performed the study selection, data extraction, and quality assessment. Studies were pooled using random-effects meta-analysis.

**Results:**

This meta-analysis included 25 high-quality studies from 12 countries, with a pooled sample of 632,368 patients with sarcoidosis. The prevalence of SAPH by transthoracic echocardiography in Europe, the United States and Asia was 18.8% [95% confidence interval (CI): 11.1–26.5%], 13.9% (95% CI: 5.4–22.4%) and 16.2% (95% CI: 7.1–25.4%) separately, and the overall pooled prevalence was 16.4% (95%CI: 12.2–20.5%). By right heart catheterization (RHC), the pooled prevalence of SAPH was 6.4% (95% CI: 3.6–9.1%) in general sarcoidosis population, and subgroup analyses showed that the prevalence of SAPH was 6.7% (95% CI: 2.4–11.0%) in Europe and 8.6% (95% CI: −4.1 to 21.3%) in the United States. Further, the prevalence of pre-capillary PH was 6.5% (95% CI: 2.9–10.2%). For the population with advanced sarcoidosis, the pooled prevalence of SAPH and pre-capillary PH by RHC was as high as 62.3% (95% CI: 46.9–77.6%) and 55.9% (95% CI: 20.1–91.7%), respectively. Finally, the pooled prevalence of SAPH in large databases with documented diagnoses (6.1%, 95% CI: 2.6–9.5%) was similar to that of RHC. Substantial heterogeneity across studies was observed for all analyses (*I*^2^ > 80%, *P* < 0.001).

**Conclusions:**

The sarcoidosis population has a relatively low burden of PH, mainly pre-capillary PH. However, as the disease progresses to advanced sarcoidosis, the prevalence of SAPH increases significantly.

## Introduction

Sarcoidosis is an inflammatory disease characterized by multisystem non-caseating granulomas with unknown causes, which most commonly affects the lungs and its surrounding lymph nodes but can also involve other organs, including the liver, kidneys, brain, heart, eyes, skin, and sinuses (1). The prevalence of sarcoidosis and its clinical presentation vary greatly according to patient sex, age group, ethnicity, and geographical region (2, 3). Consistently, the incidence of sarcoidosis is the highest among African Americans and lowest among Asians (4). Many patients with sarcoidosis have favorable outcomes, with a significant proportion showing spontaneous remission without systemic therapy. However, a small number of cases progress to advanced sarcoidosis, which is the end-stage of sarcoidosis associated with significant mortality (5, 6). The mortality rate of sarcoidosis is ~5% (5, 7).

Pulmonary hypertension (PH) is a hemodynamic and pathophysiological state characterized by an increase in the mean pulmonary arterial pressure (≥25 mmHg); a subgroup of pre-capillary PH is defined by an additional criterion of a pulmonary arterial wedge pressure of ≤ 15 mmHg (8). PH is a well-recognized complication of sarcoidosis, resulting in poor prognosis (9–11). The development of sarcoidosis-associated PH (SAPH) confers up to a 10-fold increase in mortality in patients with sarcoidosis (7, 12). SAPH is also an independent cause of death in patients with advanced pulmonary sarcoidosis (5). Therefore, it is important to know the actual prevalence of PH in patients with sarcoidosis for timely screening and treatment.

To date, the actual prevalence of SAPH is unknown; however, the condition is associated with the stage at which patients are assessed for PH. In recent years, researchers have been interested in the prevalence of SAPH and extensive related studies have been conducted worldwide. However, the results are somewhat different for the limited sample sizes and these studies are individually underpowered to effectively address this issue. Although there are relevant literatures describing and discussing this topic (13, 14), there are no meta-analysis reporting the epidemiology of SAPH globally. Therefore, we performed a systematic review and meta-analysis to comprehensively calculate the prevalence of SAPH in general and advanced sarcoidosis populations.

## Methods

### Data Source and Search Strategy

This systematic review and meta-analysis followed the Meta-analysis of Observational Studies in Epidemiology (MOOSE) (Supplementary Table 1) (15) and Preferred Reporting Items for Systematic Review and Meta-Analyses (PRISMA) guidelines (Supplementary Table 2) (16) guidelines. We performed a systematic literature search of the PubMed, Embase, and Cochrane Library databases up to July 6, 2021. The search strategy was as follows: (sarcoidosis) AND [(pulmonary hypertension) OR (pulmonary arterial hypertension) OR (PH) OR (PAH)]. The language of the searched papers was English. All reference lists of the included studies were manually searched for additional studies. Moreover, we conducted a web-based search in Internet search engines (such as Baidu Scholar and Google Scholar). As the current meta-analysis was based on previously published studies, no ethical approval or patient consent was required.

### Inclusion and Exclusion Criteria

The inclusion criteria were as follows: (1) observational study, including cross-sectional, prospective, and retrospective studies; (2) study involving the prevalence of SAPH (including pre-capillary PH and post-capillary PH); and (3) study providing the total number of sarcoidosis cases to calculate the standard error. The exclusion criteria were as follows: (1) missing essential information; (2) duplicated data used in different studies; and (3) reviews, meta-analyses, *in vitro* studies, and study protocols.

### Data Extraction

Two authors (Zhang and Tong) used a pre-designed data extraction form to independently extract data from all eligible studies. A third investigator intervened when there was any disagreement or doubt. We extracted the following information: first author; year of publication; country; sample size; number of SAPH cases identified by transthoracic echocardiography (TTE); number of SAPH cases identified by right heart catheterization (RHC); and patient age, sex, ethnicity, smoking status, and sarcoidosis stage. Notably, in the included studies, if the probability of PH was judged to be high, intermediate, or low (or the PH severity was classified as mild, moderate, and severe), the high and intermediate probabilities (or moderate and severe PH) were defined as the presence of PH. The diagnostic criteria of SAPH in included studies are shown in Supplementary Table 3. Advanced disease is mainly for patients who are at risk for death or loss of organ function (5), so in our analysis, advanced sarcoidosis refers mainly to patients with Stage IV sarcoidosis, those for lung transplantation, and those with severe life-threatening symptoms. In the study that only provided medians and ranges for several groups, we transformed the data into mean and standard deviation (SD) according to the validated methods described by Wan et al. (17), and then combined the mean and SD of multiple groups. When necessary, we also requested further information from the corresponding authors of the original studies.

### Quality Assessments

For the quality assessments, two authors (Zhang and Tong) independently assessed the risk of bias of the included studies using an adapted risk of bias tool for prevalence studies (Supplementary Table 4) (18). Selection, non-response, measurement, and analysis biases were assessed using this tool. The possible answers for every item were “low risk” or “high risk,” which were scored as “0” or “1.” Scores of 0–3, 4–6, and 7–10 were defined as low, moderate, and high risk of bias, respectively. Any disagreement was clarified and confirmed by a third investigator.

### Data Analysis and Statistical Methods

In this study, all analyses were performed using Stata 12.0, with statistical significance set at *P* < 0.05. We applied a random-effects model to obtain a pooled prevalence and a corresponding 95% confidence interval (CI) from various studies. Heterogeneity was tested using χ2 and *I*^2^ tests. A *P* < 0.10 suggested significant between-study heterogeneity. Thresholds for the interpretation of *I*^2^ were as follows: 0– 40%: might not be important; 30–60%: may represent moderate heterogeneity; 50–90%: may represent substantial heterogeneity; 75–100%: considerable heterogeneity (19). Sensitivity analyses were conducted to explore the sources of heterogeneity of results across studies by sequentially excluding eligible studies. Publication bias was assessed by Egger's and Begg's tests, with *P* < 0.05, indicating potential bias. A trim-and-fill analysis was performed to identify possible asymmetry and assess the robustness of the conclusions. Publication bias was not assessed when fewer than 10 studies were included in our analysis. If there was significant heterogeneity, subgroup analysis was further performed.

## Results

### Study Characteristics

**Figure 1:**
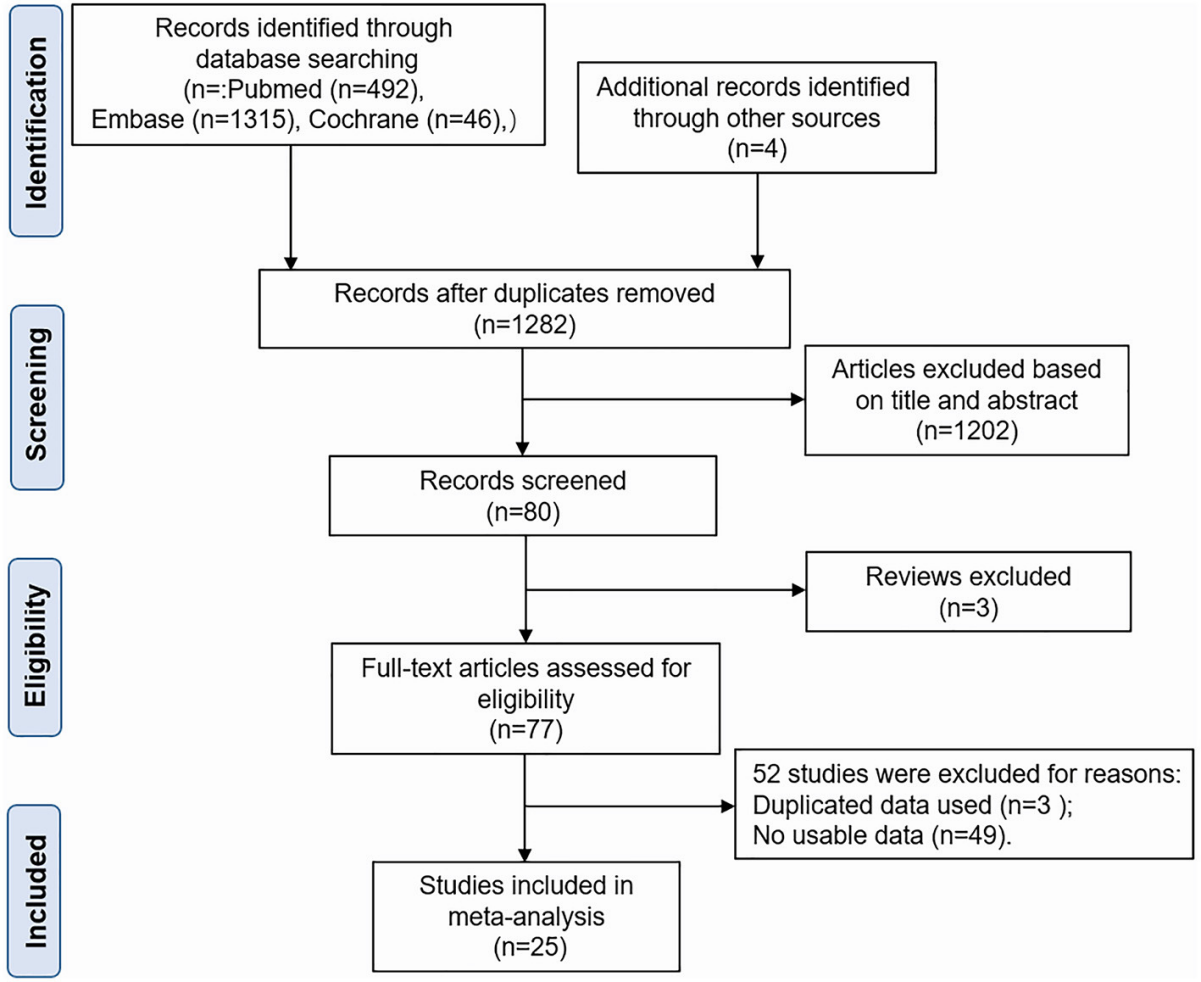
Flow diagram of the selection of studies for inclusion.

As shown in Figure 1, the initial literature search in the PubMed, Embase, and Cochrane Library databases and other sources identified 1,857 studies. After checking for duplicates, 575 studies were excluded. And 1,202 studies were removed after title and abstract review. Furthermore, three articles were excluded because they were reviews. The remaining 77 studies were further assessed for eligibility through a full-text review. Of these, 49 studies were subsequently excluded for a lack of usable data. Three studies were removed for duplicated data used in another study (13, 20, 21). Finally, the analysis included 25 studies (7, 11, 12, 22–43). The studies were conducted in the United States (*n* = 10), Europe (*n* = 9), and Asia (*n* = 6). Of these, 14 studies reported the prevalence of SAPH by TTE, 11 studies reported the prevalence of SAPH by RHC, and eight studies reported the prevalence of pre-capillary PH by RHC. Furthermore, four other studies were conducted using large databases with only documented records for the diagnosis of sarcoidosis and PH (25, 26, 28, 29). In addition, five studies included patients with advanced sarcoidosis (11, 12, 27, 37, 43); the others included the general sarcoidosis population. The basic characteristics of the included studies are shown in Table 1. The quality score of all included studies was 1–3 points, and all studies were deemed to have low risks of selection, non-response, measurement, and analysis biases (Supplementary Table 5).

**Table 1:** Characteristics of included studies.

**Study**	**Country**	**Sarcoidosis**					**SAPH**						
		***N***	**Age**	**Sex (M/F)**	**Ethnicity**	**Smoking (yes/no)**	**SAPH by TTE (*N*)**	**SAPH by RHC (*N*)**	**Pre-capillary PH by RHC (N)**	**SAPH from database (*N*)**	Age^*^	Sex^*^ (M/F)	Sarcoidosis^*^ stage (0/1/2/3/4)
Pabst et al. (34)	Germany	111	52.2 ± 14.9	65/56	NR	NR	23	5	4	N/A	66.25 ± 7.80	NR	0/0/0/3/1
Shorr et al. (43)	USA	363	46	127/236	71.6% A-A	NR	NR	268	NR	N/A	46.5 ± 7.8	97/171	NR
Huitema et al. (24)	Netherlands	479	NR	NR	NR	NR	42	17	NR	N/A	58.7 ± 12.9	13/4	NR
Rapti et al. (33)	Greece	313	54.08 ± 13.39	121/192	NR	110/203	37	9	NR	N/A	NR	NR	NR
Milman et al. (37)	Demark	24	NR	16/8	Danish	NR	NR	19	18	N/A	46.61 ± 6.87	14/5	0/0/3/0/16
Sulica et al. (42)	USA	354	NR	NR	NR	NR	54	NR	NR	N/A	50.3 ± 1.6	17/37	2/2/7/4/23
Handa et al. (40)	Japan	212	57.67 ± 14.30	55/157	Japanese	46/166	12	NR	NR	N/A	58.9 ± 13.0	7/5	2/3/1/4/2
Maimon et al. (35)	Israel	127	56.70 ± 13.46	37/91	NR	39/88	36	NR	NR	N/A	64.3 ± 11	12/25	1/5/13/17/0
Baughman et al. (12)	USA	130	53.04 ± 11.43	39/91	50.77% white	NR	NR	70	50	N/A	52 (24–76)	17/33	1/7/10/7/25
Gangemi et al. (27)	USA	28	59.23 ± 6.35	13/15	78.57% black	NR	NR	11	NR	N/A	NR	NR	NR
Kirkil et al. (7)	USA	452	50 (25–78)	139/313	68.8% white; 30.1% A-A	NR	NR	NR	29	N/A	NR	NR	NR
Huitema et al. (32)	Netherlands	89	51.69 ± 11.19	60/29	NR	NR	37	25	NR	N/A	55.8 ± 9.0	16/9	0/0/0/0/25
Smedema et al. (30)	Netherlands	87	53.27 ± 10.04	57/30	NR	NR	15	NR	NR	N/A	NR	NR	NR
Baughman et al. (39)	USA	142	51 (26–81)	41/101	61.97% A-A	NR	NR	NR	14	N/A	NR	NR	NR
Nardi et al. (11)	France	111	NR	NR	NR	NR	33	NR	NR	N/A	NR	NR	NR
Mirsaeidi et al. (31)	USA	108	NR	NR	NR	NR	6	NR	NR	N/A	NR	NR	NR
Baughman et al. (41)	USA	1,223	NR	370/853	56.50% white, 43.50% A-A	NR	NR	30	25	N/A	NR	NR	NR
Bourbonnais et al. (38)	USA	162	47 ± 12	38/124	88.3% A-A, 11.7% White	23/139	35	25	22	N/A	NR	NR	NR
Alhamad et al. (36)	Saudi Arabia	96	50.47 ± 13.75	32/64	NR	NR	20	NR	NR	N/A	49.2 ± 14.2	3/17	0/2/6/3/9
Utpat et al. (22)	India	68	42.7	27/41	NR	NR	9	NR	NR	N/A	NR	NR	NR
Özen et al. (23)	Turkish	55	52.7 ± 10.1	10/45	NR	6/49	8	3	0	N/A	64 ± 2.646	0/3	0/1/0/0/2
Tiosano et al. (25)	Israel	3,993	64.2 ± 15.7	1,471/2,522	NR	1,342/2,651	N/A	N/A	N/A	269	NR	NR	NR
Serrano et al. (26)	Spain	5,484	60.62 ± 16.28	2,395/3,089	NR	NR	N/A	N/A	N/A	337	NR	NR	NR
Frank et al. (28)	Germany	9,106	55.4 ± 15.5	4,288/4,818	NR	NR	N/A	N/A	N/A	254	NR	NR	NR
Patel et al. (29)	USA	609,051	55 ± 14	199,769/409,282	43.9% white, 49.5% black	NR	N/A	N/A	N/A	52,442	NR	NR	NR

*NR, not reported; N/A, not applicable; A-A, African-American*.

* *Data were extracted in the priority order of pre-capillary PH by RHC, SAPH by RHC, and SAPH by TTE*.

### Prevalence of Sarcoidosis-Associated Pulmonary Hypertension by Transthoracic Echocardiography

**Figure 2:**
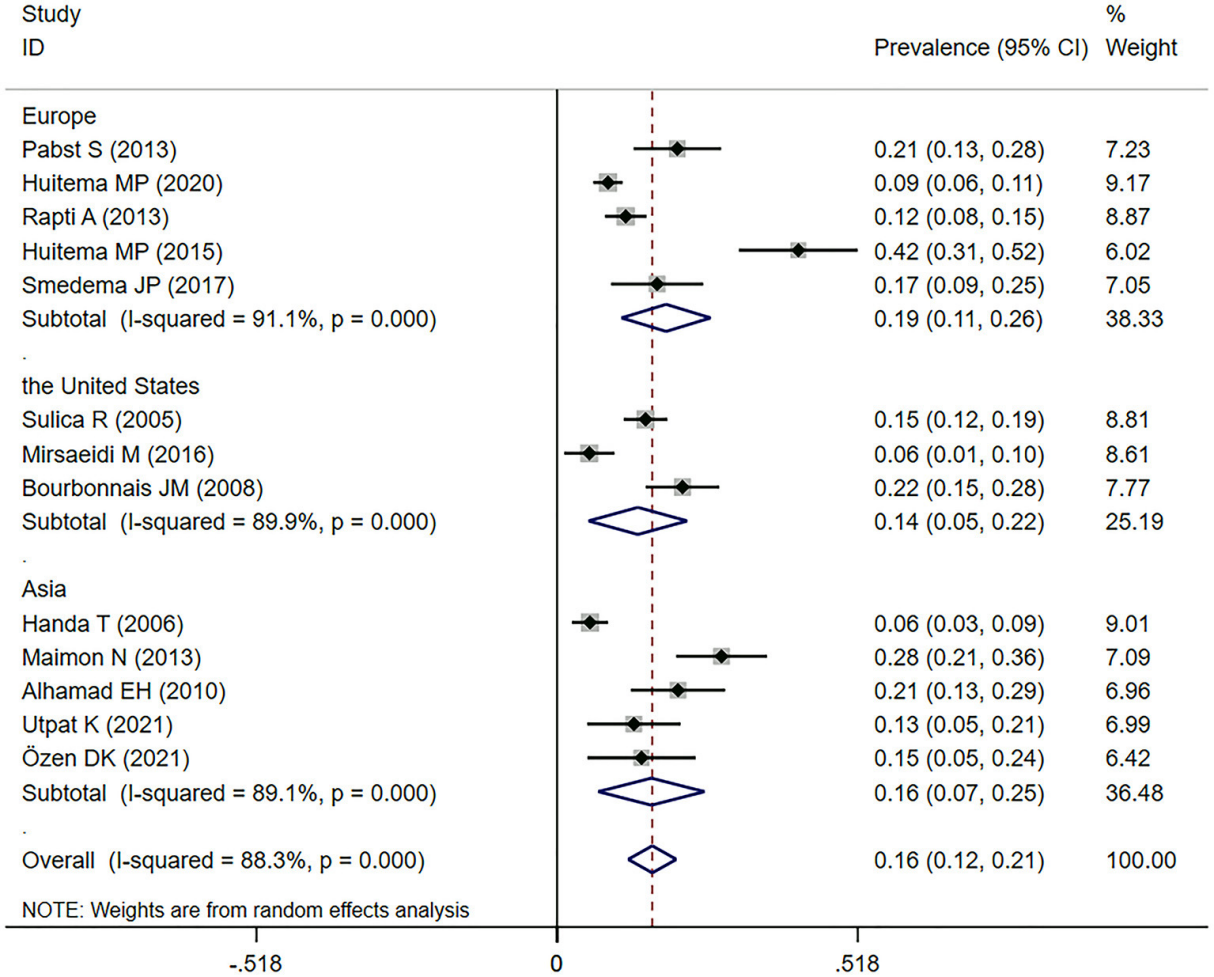
Forest plot of the pooled prevalence of SAPH by TTE in the general sarcoidosis population. SAPH, sarcoidosis-associated pulmonary hypertension; TTE, transthoracic echocardiography.

Fourteen studies (11, 22–24, 30–36, 38, 40, 42) reported the prevalence of SAPH by TTE. Among them, one study targeted a population with advanced sarcoidosis, among which the prevalence of SAPH by TTE was 29.7% (11). In the general sarcoidosis population, the prevalence of SAPH by TTE in Europe, the United States, and Asia were 18.8% (95% CI: 11.1–26.5%), 13.9% (95% CI: 5.4–22.4%), and 16.2% (95% CI: 7.1–25.4%), separately. Overall, 334 of 2,261 sarcoidosis patients had SAPH by TTE and the pooled prevalence of SAPH by TTE was 16.4% (95% CI: 12.2–20.5%) (Figure 2). However, the heterogeneity between studies was substantial (*I*^2^ = 88.3%, *P* < 0.001). A sensitivity analysis to explore the effect of each study on the pooled meta-results showed no substantial changes in the pooled prevalence, indicating the stability of our meta-analysis (Supplementary Figure 1). In terms of publication bias, Begg's and Egger's tests revealed *P*-values of 0.059 and 0.003, respectively, indicating some publication bias. However, there was no indication of publication bias by trim-and-fill method (no trimming was performed, and the data were unchanged) (Supplementary Figure 2).

### Prevalence of Sarcoidosis-Associated Pulmonary Hypertension by Right Heart Catheterization

**Figure 3:**
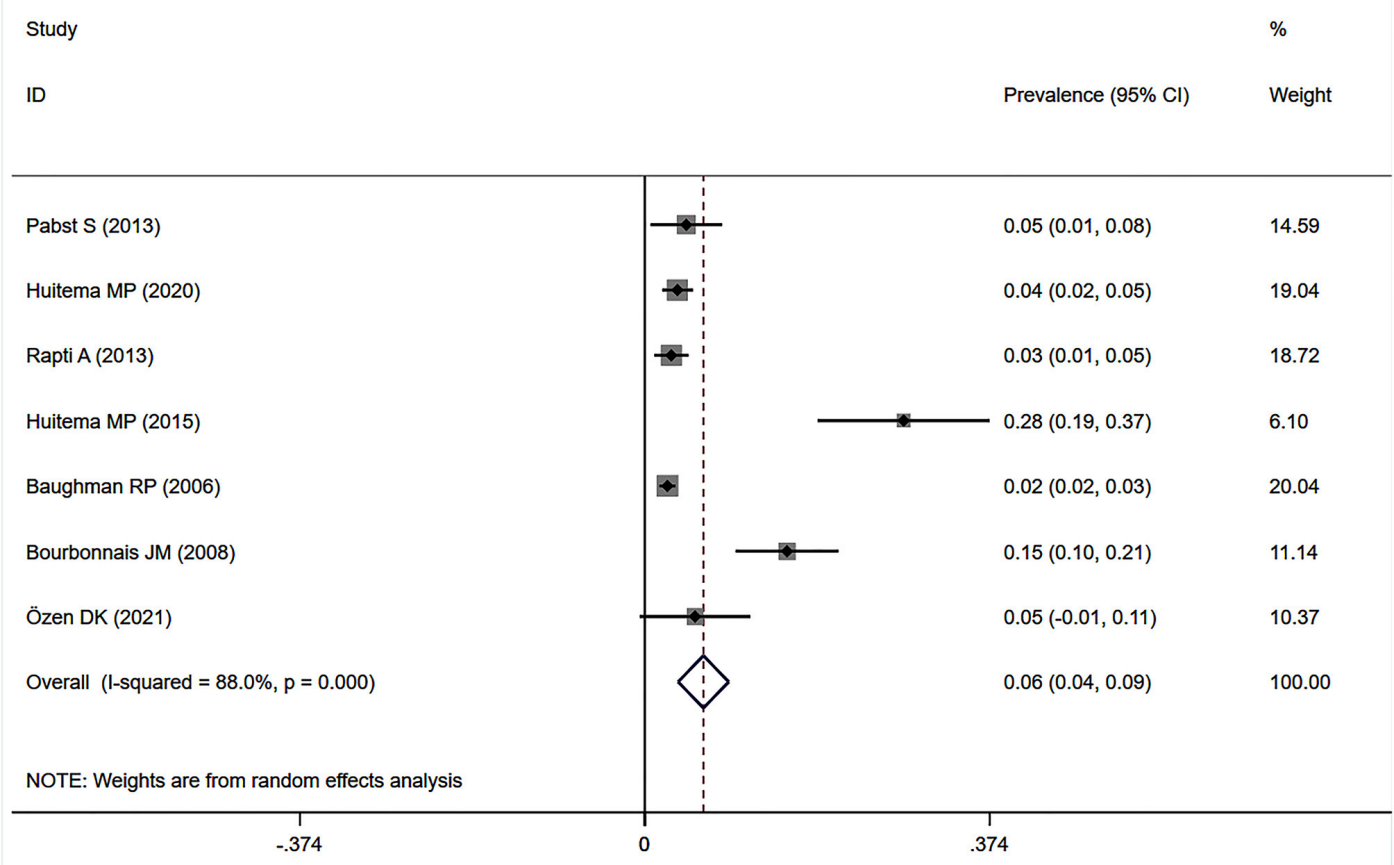
Forest plot of the pooled prevalence of SAPH by RHC in general sarcoidosis population. SAPH, sarcoidosis-associated pulmonary hypertension; RHC, right heart catheterization.

**Figure 4:**
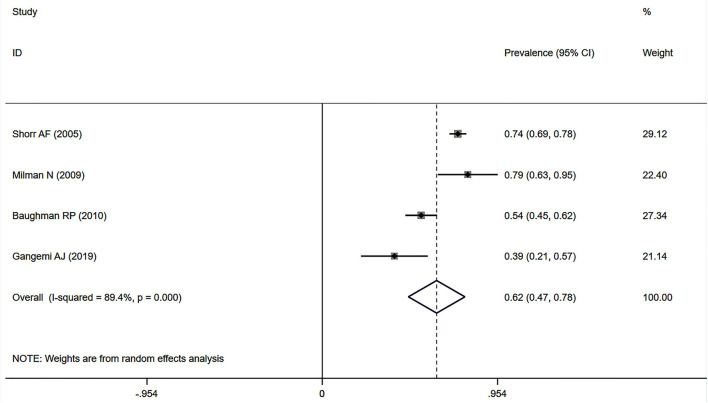
Forest plot of the pooled prevalence of SAPH by RHC in advanced sarcoidosis population. SAPH, sarcoidosis-associated pulmonary hypertension; RHC, right heart catheterization.

Eleven studies (12, 23, 24, 27, 32–34, 37, 38, 41, 43) described the prevalence of SAPH by RHC. Of these, seven studies focused on the general sarcoidosis population, while four studies focused on the population with advanced sarcoidosis. In 2,432 general sarcoidosis patients, 114 had SAPH by RHC, with an estimated prevalence of SAPH by RHC of 6.4% (95% CI: 3.6–9.1%) (Figure 3). Among the population with advanced sarcoidosis, the estimated prevalence of SAPH by RHC was 62.3% (95% CI: 46.9–77.6%) (Figure 4). Significant heterogeneity across studies was observed for both the general (*I*^2^ = 88.0%, *P* < 0.001) and advanced sarcoidosis (*I*^2^ = 89.4%, *P* < 0.001) populations. Sensitivity analysis revealed that our meta-results were stable in both groups (Supplementary Figures 3, 4).

**Figure 5:**
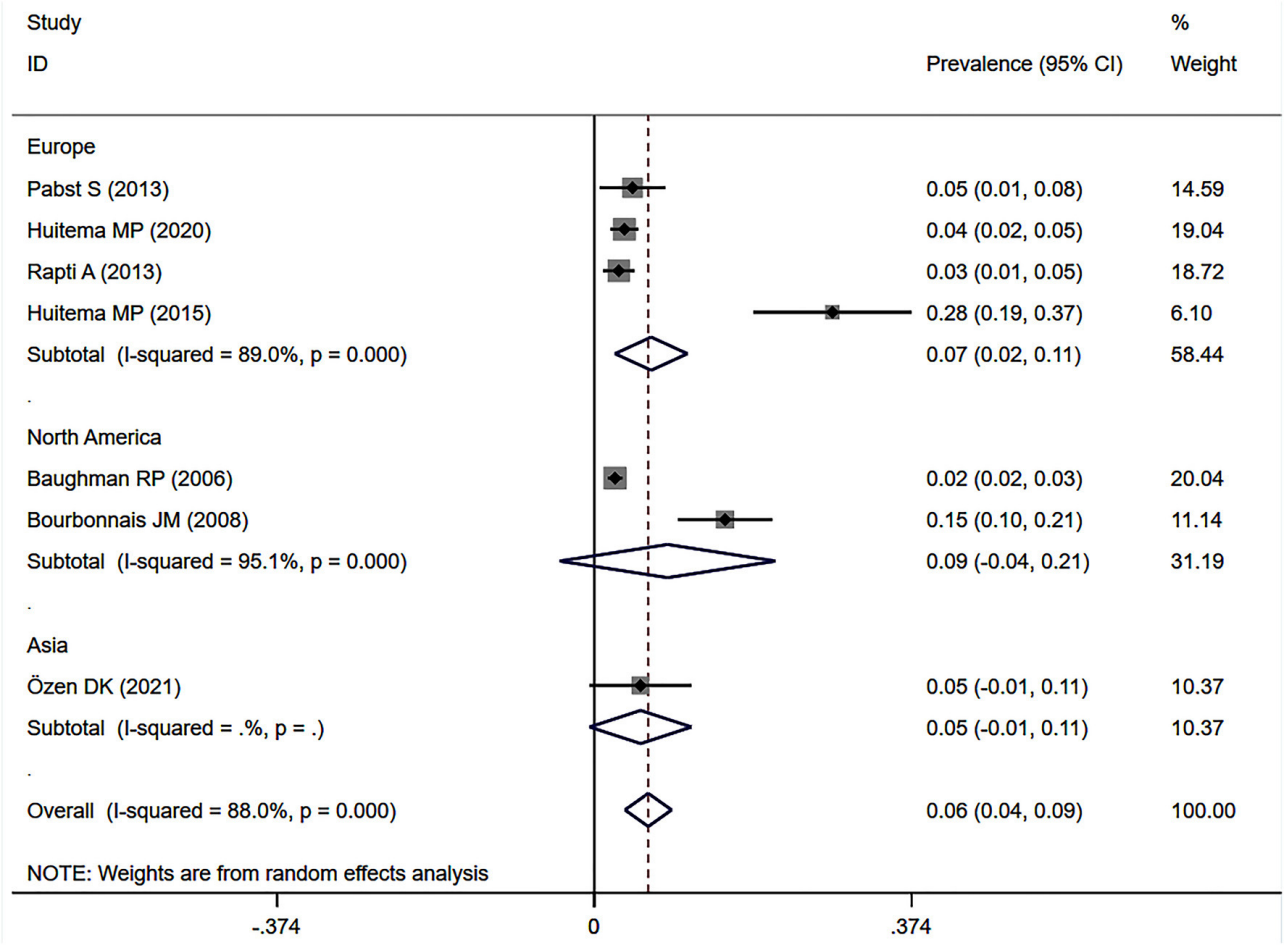
Subgroup analysis of the prevalence of SAPH by RHC in the general sarcoidosis population by geographical regions. SAPH, sarcoidosis-associated pulmonary hypertension; RHC, right heart catheterization.

Subgroup analyses by geographical region showed persisting heterogeneity. The pooled prevalence of SAPH by RHC was 6.7% (95% CI: 2.4–11.0%) in Europe and 8.6% (95% CI: −4.1 to 21.3%) in the United States for the general sarcoidosis population (Figure 5).

### Prevalence of Sarcoidosis Associated Pre-capillary Pulmonary Hypertension by Right Heart Catheterization

**Figure 6:**
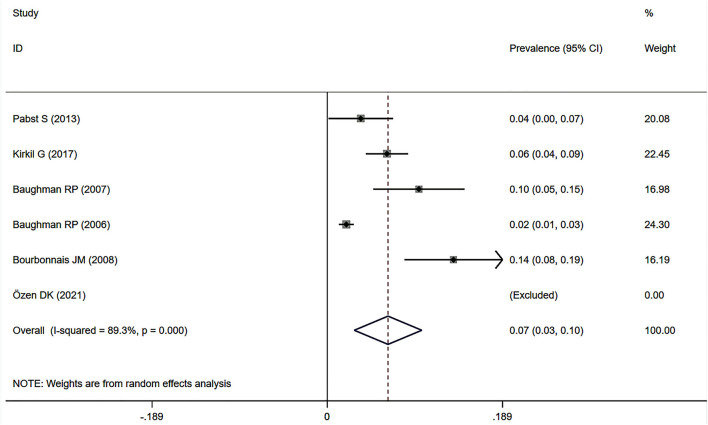
Forest plot of the pooled prevalence of pre-capillary PH by RHC in general sarcoidosis population. PH, pulmonary hypertension; RHC, right heart catheterization.

**Figure 7:**
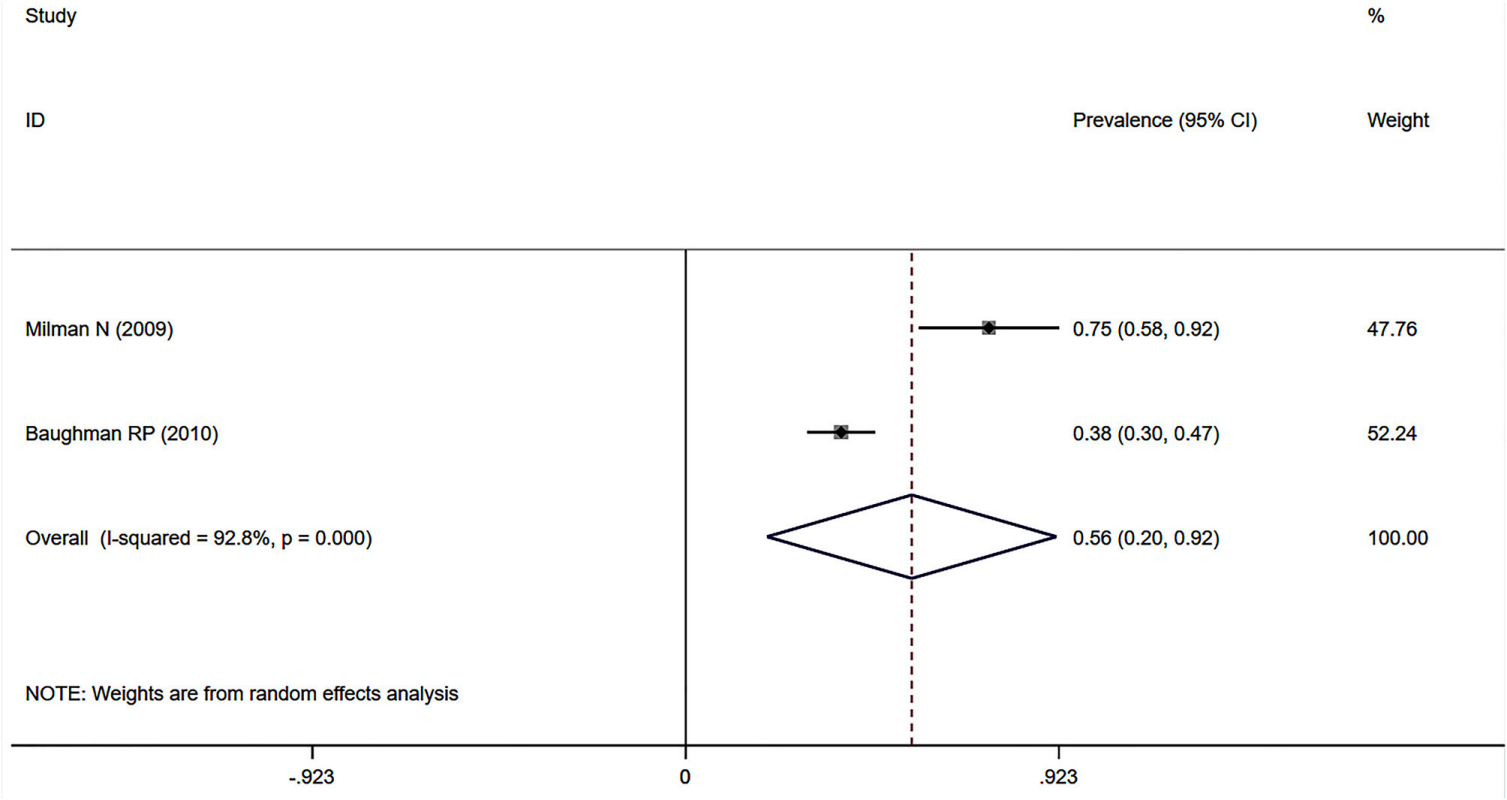
Forest plot of the pooled prevalence of pre-capillary PH by RHC in the population with advanced sarcoidosis. PH, pulmonary hypertension; RHC, right heart catheterization.

Eight studies (7, 12, 23, 34, 37–39, 41) showed the prevalence of pre-capillary PH by RHC in patients with sarcoidosis, six of which focused on the general sarcoidosis population. In the general sarcoidosis population, the estimated prevalence of pre-capillary PH by RHC was 6.5% (95% CI: 2.9–10.2%) (Figure 6), with a high degree of heterogeneity across studies (*I*^2^ = 89.3%, *P* < 0.001). Sensitivity analysis revealed that our meta-results were stable (Supplementary Figure 5). In addition, two studies reported the prevalence of advanced sarcoidosis, with a pooled prevalence of pre-capillary PH by RHC of 55.9% (95% CI: 20.1–91.7%) (Figure 7).

### Prevalence of Sarcoidosis-Associated Pulmonary Hypertension in Large Databases With Documented Records for Diagnoses

**Figure 8:**
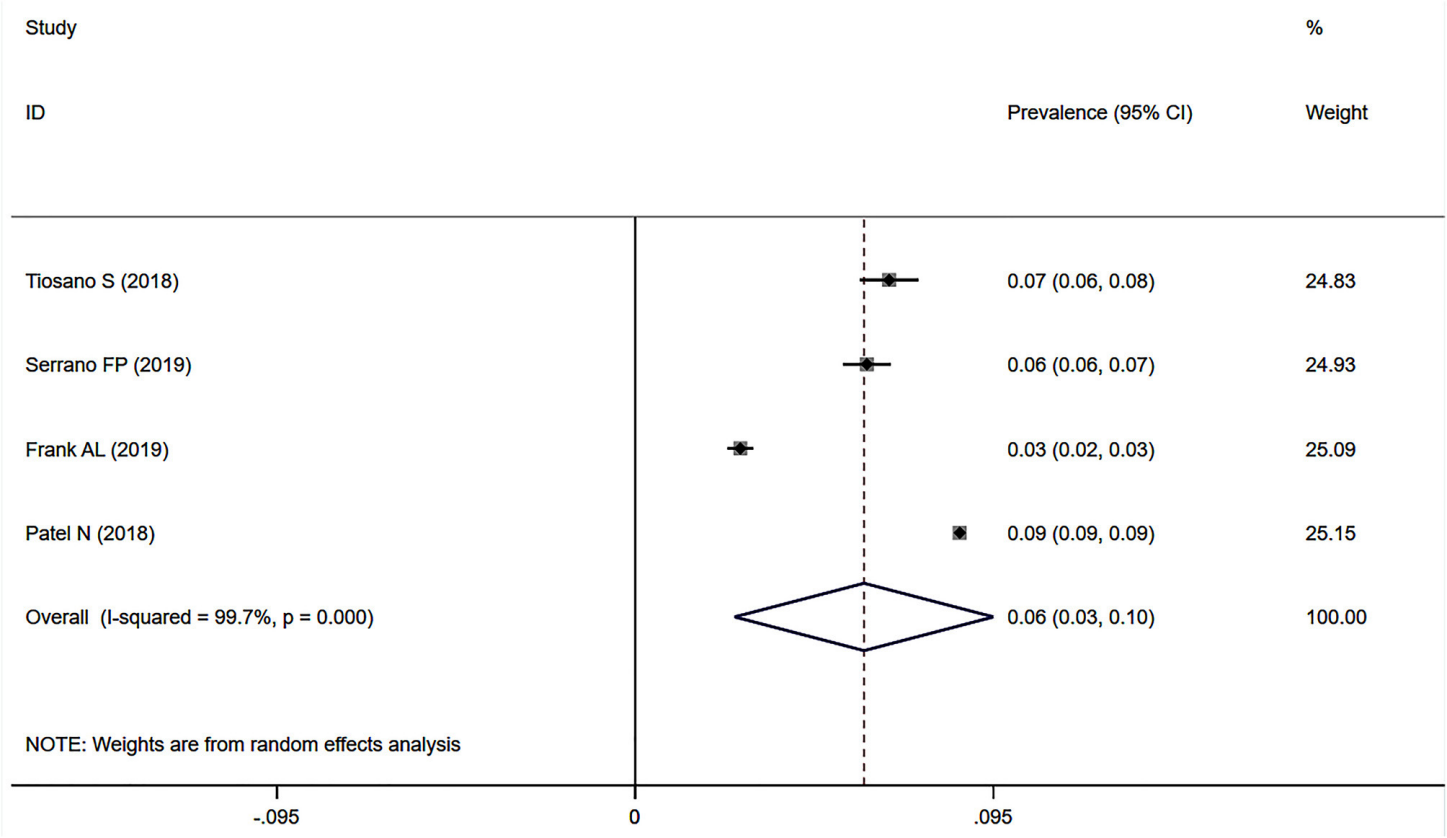
Forest plot of the pooled prevalence of SAPH in large databases with documented diagnoses. SAPH, sarcoidosis-associated pulmonary hypertension.

Four studies (25, 26, 28, 29) used data on the prevalence of SAPH in large databases with documented records for diagnoses of sarcoidosis and PH (i.e., the chronic disease registry of Clalit Health Services in Israel, the Spanish National Hospital Discharge Database, the patient-individual health insurance claims data in Germany, and the National Inpatient Sample database in the United States). Among 627,634 sarcoidosis records, 53,302 had PH. The pooled prevalence of SAPH was 6.1% (95% CI: 2.6–9.5%) (Figure 8), with a non-negligible heterogeneity (*I*^2^ = 99.7%, *P* < 0.001). Sensitivity analysis revealed the stability of our meta-analysis results (Supplementary Figure 6).

## Discussion

Sarcoidosis can affect multiple organs, including the lungs, liver, kidneys, brain, heart, eyes, skin, and sinuses, and can cause a variety of complications. Among them, cardiovascular complications, such as conduction abnormalities, arrhythmias, heart failure, and PH, are associated with higher mortality (14, 44, 45). SAPH is closely associated with poor prognosis, conferring up to a 10-fold increase in mortality in patients with sarcoidosis. Therefore, estimating the prevalence of SAPH is critical to the treatment and prognosis of sarcoidosis and essential for adequate health services planning and organization.

This is the first comprehensive meta-analysis to estimate the prevalence of SAPH in patients with sarcoidosis. Twenty-five high-quality studies from 12 countries met the inclusion criteria, with a pooled sample of 632,368 patients with sarcoidosis. In the general sarcoidosis population, the pooled prevalence of SAPH was 16.4% by TTE and 6.4% by RHC but was as high as 62.3% by RHC in the population with advanced sarcoidosis. Furthermore, the estimated prevalence of pre-capillary PH by RHC was 6.5% in the general sarcoidosis population and 55.9% in the population with advanced sarcoidosis. The prevalence of SAPH in large databases with documented diagnoses was 6.1%.

The mechanisms of PH in sarcoidosis patients are unclear and multifactorial. The possible triggers include progressive pulmonary fibrosis and hypoxic vasoconstriction (46), small vessel inflammatory vasculopathy (46, 47), diffuse alveolar-capillary multiplication (48, 49), pulmonary veno-occlusive disease (50), chronic thromboembolism and non-thromboembolic pulmonary embolism (51–53), extrinsic compression of pulmonary vessels caused by lymphadenopathy (54), myocardial involvement (55), sleep-disordered breathing (56), and liver dysfunction causing portopulmonary hypertension and anemia (14). Many confounders also affect the prevalence of SAPH, particularly the stage at which sarcoidosis patients are assessed (14). In our study, SAPH occurred much more frequently in patients with advanced sarcoidosis.

Surprisingly, our meta-analysis showed that the prevalence of pre-capillary PH by RHC (6.5%) was similar to that for SAPH by RHC (6.4%), or even slightly higher in general sarcoidosis patients, mainly because the studies included in each analysis were not completely consistent. To address this problem, we performed a meta-analysis of four studies (23, 34, 38, 41) that reported both the prevalence of PH and pre-capillary PH by RHC for general sarcoidosis patients, the results of which showed that the prevalence of SAPH by RHC (6.5%) (Supplementary Figure 7) was higher than that of pre-capillary PH (5.8%) (Supplementary Figure 8). In all included studies, only two studies (31, 33) excluded patients with cardiac dysfunction. Thus, our results showed that SAPH was mostly pre-capillary PH to some extent, with few parts related to cardiovascular conditions (mainly left heart disease) (57).

We also estimated the prevalence of SAPH in large databases with documented records for diagnoses. Surprisingly, the prevalence of SAPH in the large databases (6.1%) was similar to that by RHC (6.4%). These results indicated that the clinical diagnosis of SAPH mainly depends on RHC rather than TTE. The higher prevalence of PH by TTE than that by RHC is mainly based on the peak tricuspid regurgitation velocity or an estimated systolic pulmonary arterial pressure, which can only assign the echocardiographic probability of PH. Finally, the gold standard for the diagnosis of PH is RHC (8). When interpreted in a clinical context, TTE should always be performed initially in cases of suspected PH because TTE is non-invasive and cheaper, and if there are indications of PH, RHC should be considered to confirm the diagnosis of PH. In addition, TTE can help detect the cause of PH.

The presence of SAPH is an independent risk factor for poor prognosis in patients with sarcoidosis (46, 58, 59). As this meta-analysis aimed to calculate the prevalence of SAPH to inform clinic settings, attention should be paid to therapy after the confirmation of SAPH. The pathophysiology of SAPH is relevant to individual treatment. Currently, the approved medical therapies for PH are mainly for patients with group 1 pulmonary arterial hypertension (PAH); however, SAPH may be somewhat similar to PAH (54). Studies on PAH-directed therapies in the treatment of SAPH have demonstrated that pulmonary vasodilators (mainly referred to as endothelin receptor antagonists, phosphodiesterase inhibitors, and prostacyclins) can improve hemodynamics and functional status (21, 60–64). Additional treatments for SAPH include combination therapy with pulmonary vasodilators and immunomodulation, diuretics for volume optimization, stenting for mechanical vascular obstruction, pulmonary endarterectomy, balloon pulmonary angioplasty, and even lung transplantation (14, 54).

In our meta-analysis, the heterogeneity between studies was substantial, with many analyses showing *I*^2^ > 80%. Previous meta-analyses on prevalence studies have reported similar results (65, 66). The subgroup analyses failed to determine the reasons for this significant heterogeneity. There was insufficient information or quantity of studies to conduct subgroups according to sex, smoking, and the stage of sarcoidosis. The incidence of sarcoidosis in different races is different and evaluating the impact of ethnicity on SAPH is of significance. However, few included studies reported information on ethnicity and detailed data on the prevalence of SAPH in different races were not available. Thus, this study could not conduct a subgroup analysis by ethnicity. SAPH is also related to the severity of lung disease and chronic hypoxemia, but there was insufficient information to analyze the severity of PH based on current published literature. In addition, the inconsistent use of TTE and RHC to confirm the diagnosis of PH in all included studies contributed to the heterogeneity.

This study has several strengths. First, this was the first meta-analysis to estimate the prevalence of SAPH. Second, the 25 high-quality included studies from 12 countries provided a sufficient sample size with a pooled sample of 632,368 patients with sarcoidosis, which is powered to effectively address this issue. Third, our study assessed the prevalence of SAPH from multiple perspectives, including TTE, RHC, and data from a large database with documented records for diagnoses, as well as the prevalence of pre-capillary PH (a subgroup of PH) in patients with sarcoidosis. Nevertheless, this study also has some limitations arising from the included studies. First, because the prevalence of SAPH was not the main focus but was rather integrated into some studies, the reporting was often suboptimal for the purpose of this meta-analysis. Second, our meta-analysis identified significant publication bias, particularly regarding the prevalence of SAPH by TTE, which might be associated with the small sample sizes in most of the included studies and the English language restriction during study screening. Third, the included studies were concentrated in the United States (*n* = 10), Europe (*n* = 9), and Asia (*n* = 6), which might affect the generalizability of the findings. Finally, some analyses included small numbers of studies, resulting in limited statistical confidence. Despite these limitations, we were able to minimize bias throughout the entire analysis process.

## Conclusions

Estimating the prevalence of SAPH is essential for adequate planning and organizing health services. Based on the available literature, the pooled prevalence of SAPH was 16.4% by TTE and 6.4% by RHC in the general sarcoidosis population but was as high as 62.3% by RHC in the population with advanced sarcoidosis. The estimated prevalence of pre-capillary PH by RHC was 6.5% in the general sarcoidosis population and 55.9% in the population with advanced sarcoidosis. To yield more accurate prevalence estimates, more high-quality cohort studies are warranted and procedures and prevalence studies should be standardized.

## Data Availability Statement

The original contributions presented in the study are included in the article/Supplementary Material, further inquiries can be directed to the corresponding author/s.

## Author Contributions

HF and XT conceived of the study. SZ, XT, and TZ performed the literature search and selection, data extraction, and assessment of risk of bias. SZ, TZ, DW, SL, and LW carried out the data analysis. SZ drafted the manuscript. All authors critically revised the article and approved the final draft for publication.

## Funding

This study was supported by China Postdoctoral Science Foundation (2020M673259), Post-Doctor Research Project, West China Hospital, Sichuan University (2020HXBH013), and 1•3•5 project for disciplines of excellence–Clinical Research Incubation Project, West China Hospital, Sichuan University (2019HXFH008).

## Conflict of Interest

The authors declare that the research was conducted in the absence of any commercial or financial relationships that could be construed as a potential conflict of interest.

## Publisher's Note

All claims expressed in this article are solely those of the authors and do not necessarily represent those of their affiliated organizations, or those of the publisher, the editors and the reviewers. Any product that may be evaluated in this article, or claim that may be made by its manufacturer, is not guaranteed or endorsed by the publisher.

## Supplementary Material

Supplementary Figure 1 Results of the sensitivity analysis of the prevalence of SAPH by TTE. SAPH, sarcoidosis-associated pulmonary hypertension; TTE, transthoracic echocardiography.

Supplementary Figure 2 Results of the trim-and-fill analysis of SAPH by TTE in the general sarcoidosis population. SAPH, sarcoidosis-associated pulmonary hypertension; TTE, transthoracic echocardiography.

Supplementary Figure 3 Results of the sensitivity analysis of the prevalence of SAPH by RHC in the general sarcoidosis population. SAPH, sarcoidosis-associated pulmonary hypertension; RHC, right heart catheterization.

Supplementary Figure 4 Results of the sensitivity analysis of the prevalence of SAPH by RHC in the population with advanced sarcoidosis. SAPH, sarcoidosis-associated pulmonary hypertension; RHC, right heart catheterization.

Supplementary Figure 5 Results of the sensitivity analysis of the prevalence of pre-capillary PH by RHC in the general sarcoidosis population. PH, pulmonary hypertension; RHC, right heart catheterization.

Supplementary Figure 6 Results of sensitivity analysis of the prevalence of SAPH based on documented diagnoses in large databases. SAPH, sarcoidosis-associated pulmonary hypertension.

Supplementary Figure 7 Forest plot of the pooled prevalence of SAPH for studies reporting both PH and pre-capillary PH. SAPH, sarcoidosis-associated pulmonary hypertension; PH, pulmonary hypertension.

Supplementary Figure 8 Forest plot of the pooled prevalence of pre-capillary PH in sarcoidosis patients for studies reporting both PH and pre-capillary PH. PH, pulmonary hypertension.

Supplementary Table 1 MOOSE checklist (15).

Supplementary Table 2 PRISMA checklist.

Supplementary Table 3 Diagnostic criteria of pulmonary hypertension in included studies.

Supplementary Table 4 Risk of bias quality assessment tool.

Supplementary Table 5 Quality assessment of included studies.

## References

[1] Raghu G Berman JS Govender P. Treatment of sarcoidosis. Am J Resp Crit Care Med. (2018) 197:P9–10. 10.1164/rccm.1976P9 29543101

[2] Zhou Y Gerke AK Lower EE Vizel A Talwar D Strambu I . The impact of demographic disparities in the presentation of sarcoidosis: a multicenter prospective study. Respir Med. (2021) 187:106564. 10.1016/j.rmed.2021.106564 34391118 PMC9999732

[3] Judson MA Boan AD Lackland DT. The clinical course of sarcoidosis: presentation, diagnosis, and treatment in a large white and black cohort in the United States. Sarcoid Vasc Diffuse Lung Dis. (2012) 29:119–27. 23461074

[4] Grunewald J Grutters JC Arkema EV Saketkoo LA Moller DR Muller-Quernheim J. Sarcoidosis. Nat Rev Dis Primers. (2019) 5:45. 10.1038/s41572-019-0096-x 31273209

[5] Baughman RP Wells A. Advanced sarcoidosis. Curr Opin Pulm Med. (2019) 25:497–504. 10.1097/MCP.0000000000000612 31365384

[6] Shlobin OA Nathan SD. Management of end-stage sarcoidosis: pulmonary hypertension and lung transplantation. Eur Respir J. (2012) 39:1520–33. 10.1183/09031936.00175511 22241743

[7] Kirkil G Lower EE Baughman RP. Predictors of mortality in pulmonary sarcoidosis. Chest. (2018) 153:105–13. 10.1016/j.chest.2017.07.008 28728933

[8] Galie N Humbert M Vachiery JL Gibbs S Lang I Torbicki A . 2015 ESC/ERS Guidelines for the diagnosis and treatment of pulmonary hypertension: the joint task force for the diagnosis and treatment of pulmonary hypertension of the European society of cardiology (ESC) and the European respiratory society (ERS): Endorsed by: association for European paediatric and congenital cardiology (AEPC), international society for heart and lung transplantation (ISHLT). Eur Respir J. (2015) 46:903–75. 10.1183/13993003.01032-2015 26318161

[9] Shlobin OA Kouranos V Barnett SD Alhamad EH Culver DA Barney J . Physiological predictors of survival in patients with sarcoidosis-associated pulmonary hypertension: results from an international registry. Eur Respir J. (2020) 55:1901747. 10.1183/13993003.01747-2019 32139456

[10] Boucly A Cottin V Nunes H Jaïs X Tazi A Prévôt G . Management and long-term outcomes of sarcoidosis-associated pulmonary hypertension. Eur Respir J. (2017) 50:1700465. 10.1183/13993003.00465-2017 29051269

[11] Nardi A Brillet PY Letoumelin P Girard F Brauner M Uzunhan Y . Stage IV sarcoidosis: comparison of survival with the general population and causes of death. Eur Respir J. (2011) 38:1368–73. 10.1183/09031936.00187410 22075486

[12] Baughman RP Engel PJ Taylor L Lower EE. Survival in sarcoidosis-associated pulmonary hypertension: the importance of hemodynamic evaluation. Chest. (2010) 138:1078–85. 10.1378/chest.09-2002 20348196

[13] Pabst S Grohé C Skowasch D. Prevalence of sarcoidosis-associated pulmonary hypertension: cumulative analysis of two PULSAR studies. Eur Respir J. (2020) 55:1902223. 10.1183/13993003.02223-2019 32051184

[14] Samaranayake CB McCabe C Wort SJ Price LC. Sarcoidosis associated pulmonary hypertension. Curr Opin Pulm Med. (2021) 27:285–95. 10.1097/MCP.0000000000000793 34127623

[15] Stroup DF Berlin JA Morton SC Olkin I Williamson GD Rennie D . Meta-analysis of observational studies in epidemiology: a proposal for reporting. Meta-analysis of observational studies in epidemiology (MOOSE) group. JAMA. (2000) 283:2008–12. 10.1001/jama.283.15.2008 10789670

[16] Moher D Liberati A Tetzlaff J Altman DG. Preferred reporting items for systematic reviews and meta-analyses: the PRISMA statement. J Clin Epidemiol. (2009) 62:1006–12. 10.1016/j.jclinepi.2009.06.005 19631508

[17] Wan X Wang W Liu J Tong T. Estimating the sample mean and standard deviation from the sample size, median, range and/or interquartile range. BMC Med Res Methodol. (2014) 14:135. 10.1186/1471-2288-14-135 25524443 PMC4383202

[18] Hoy D Brooks P Woolf A Blyth F March L Bain C . Assessing risk of bias in prevalence studies: modification of an existing tool and evidence of interrater agreement. J Clin Epidemiol. (2012) 65:934–9. 10.1016/j.jclinepi.2011.11.014 22742910

[19] Higgins J Green S. Cochrane Handbook for Systematic Reviews of Interventions Version 5.1.0 [updated March 2011]. The Cochrane Collaboration. (2011). Available online at: www.cochrane-handbook.org (accessed October, 2021).

[20] Huitema MP Bakker ALM Mager JJ Rensing B Smits F Snijder RJ . Prevalence of pulmonary hypertension in pulmonary sarcoidosis: the first large European prospective study. Eur Respir J. (2019) 54:1900897. 10.1183/13993003.00897-2019 31320453

[21] Milman N Burton CM Iversen M Videbaek R Jensen CV Carlsen J. Pulmonary hypertension in end-stage pulmonary sarcoidosis: therapeutic effect of sildenafil? J Heart Lung Transplant. (2008) 27:329–34. 10.1016/j.healun.2007.11.576 18342757

[22] Utpat K Sasikumar C Desai U Joshi JM. Sarcoidosis at the pulmonary medicine department of a tertiary care hospital in Mumbai. Our experience and the new modified criteria clinical radiological physiological (TNMC CRP) score for sarcoidosis: a novel proposition to assess the functional status. Monaldi Arch Chest Dis. (2021) 91:1636. 10.4081/monaldi.2021.1636 33594856

[23] Kaptan Ozen D Mutlu B Kocakaya D Turan B Sert Sekerci S Ceyhan B. Pulmonary hypertension in patients with sarcoidosis: a single-center experience. Anatol J Cardiol. (2021) 25:36–41. 10.14744/AnatolJCardiol.2020.88054 33382054 PMC7803809

[24] Huitema MP Bakker ALM Mager JJ Snijder RJ Rensing B Swaans MJ . Predicting pulmonary hypertension in sarcoidosis; value of PH probability on echocardiography. Int J Cardiovasc Imag. (2020) 36:1497–505. 10.1007/s10554-020-01859-9 32350704

[25] Tiosano S Versini M Dar Antaki L Spitzer L Yavne Y Watad A . The long-term prognostic significance of sarcoidosis-associated pulmonary hypertension - a cohort study. Clin Immunol. (2019) 199:57–61. 10.1016/j.clim.2018.12.012 30543925

[26] Pedraza-Serrano F Jimenez-Garcia R Lopez-de-Andres A Hernandez-Barrera V Sanchez-Munoz G Puente-Maestu L . Characteristics and outcomes of patients hospitalized with interstitial lung diseases in Spain, 2014 to 2015. Medicine. (2019) 98:e15779. 10.1097/MD.0000000000015779 31124970 PMC6571208

[27] Gangemi AJ Myers CN Zheng M Brown J Butler-LeBair M Cordova F . Mortality for sarcoidosis patients on the transplant wait list in the lung allocation score era: experience from a high volume center. Respir Med. (2019) 157:69–76. 10.1016/j.rmed.2019.09.001 31522032

[28] Frank AL Kreuter M Schwarzkopf L. Economic burden of incident interstitial lung disease (ILD) and the impact of comorbidity on costs of care. Respir Med. (2019) 152:25–31. 10.1016/j.rmed.2019.04.009 31128606

[29] Patel N Kalra R Doshi R Arora H Bajaj NS Arora G . Hospitalization rates, prevalence of cardiovascular manifestations, and outcomes associated with sarcoidosis in the United States. J Am Heart Assoc. (2018) 7:e007844. 10.1161/JAHA.117.007844 29358190 PMC5850171

[30] Smedema JP van Geuns RJ Ainslie G Ector J Heidbuchel H Crijns H. Right ventricular involvement in cardiac sarcoidosis demonstrated with cardiac magnetic resonance. ESC Heart Fail. (2017) 4:535–44. 10.1002/ehf2.12166 29154434 PMC5695200

[31] Mirsaeidi M Omar HR Baughman R Machado R Sweiss N. The association between BNP, 6MWD test, DLCO% and pulmonary hypertension in sarcoidosis. Sarcoid Vasc Diffuse Lung Dis. (2016) 33:317–20. 28079843

[32] Huitema MP Spee M Vorselaars VM Boerman S Snijder RJ van Es HW . Pulmonary artery diameter to predict pulmonary hypertension in pulmonary sarcoidosis. Eur Respir J. (2016) 47:673–6. 10.1183/13993003.01319-2015 26493790

[33] Rapti A Kouranos V Gialafos E Aggeli K Moyssakis J Kallianos A . Elevated pulmonary arterial systolic pressure in patients with sarcoidosis: prevalence and risk factors. Lung. (2013) 191:61–7. 10.1007/s00408-012-9442-4 23229755

[34] Pabst S Hammerstingl C Grau N Kreuz J Grohe C Juergens UR . Pulmonary arterial hypertension in patients with sarcoidosis: the Pulsar single center experience. Adv Exp Med Biol. (2013) 755:299–305. 10.1007/978-94-007-4546-9_38 22826080

[35] Maimon N Salz L Shershevsky Y Matveychuk A Guber A Shitrit D. Sarcoidosis-associated pulmonary hypertension in patients with near-normal lung function. Int J Tuberc Lung Dis. (2013) 17:406–11. 10.5588/ijtld.12.0428 23407231

[36] Alhamad EH Idrees MM Alanezi MO Alboukai AA Shaik SA. Sarcoidosis-associated pulmonary hypertension: clinical features and outcomes in Arab patients. Ann Thorac Med. (2010) 5:86–91. 10.4103/1817-1737.62471 20582173 PMC2883203

[37] Milman N Svendsen CB Iversen M Videbaek R Carlsen J. Sarcoidosis-associated pulmonary hypertension: acute vasoresponsiveness to inhaled nitric oxide and the relation to long-term effect of sildenafil. Clin Respir J. (2009) 3:207–13. 10.1111/j.1752-699X.2008.00120.x 20298406

[38] Bourbonnais JM Samavati L. Clinical predictors of pulmonary hypertension in sarcoidosis. Eur Respir J. (2008) 32:296–302. 10.1183/09031936.00175907 18385166

[39] Baughman RP Sparkman BK Lower EE. Six-minute walk test and health status assessment in sarcoidosis. Chest. (2007) 132:207–13. 10.1378/chest.06-2822 17625083

[40] Handa T Nagai S Miki S Fushimi Y Ohta K Mishima M . Incidence of pulmonary hypertension and its clinical relevance in patients with sarcoidosis. Chest. (2006) 129:1246–52. 10.1378/chest.129.5.1246 16685015

[41] Baughman RP Engel PJ Meyer CA Barrett AB Lower EE. Pulmonary hypertension in sarcoidosis. Sarcoid Vasc Diffuse Lung Dis. (2006) 23:108–16. 17937106

[42] Sulica R Teirstein AS Kakarla S Nemani N Behnegar A Padilla ML. Distinctive clinical, radiographic, and functional characteristics of patients with sarcoidosis-related pulmonary hypertension. Chest. (2005) 128:1483–9. 10.1378/chest.128.3.1483 16162747

[43] Shorr AF Helman DL Davies DB Nathan SD. Pulmonary hypertension in advanced sarcoidosis: epidemiology and clinical characteristics. Eur Respir J. (2005) 25:783–8. 10.1183/09031936.05.00083404 15863633

[44] Shade JK Prakosa A Popescu DM Yu R Okada DR Chrispin J . Predicting risk of sudden cardiac death in patients with cardiac sarcoidosis using multimodality imaging and personalized heart modeling in a multivariable classifier. Sci Adv. (2021) 7:eabi8020. 10.1126/sciadv.abi8020 34321202 PMC8318376

[45] Alba AC Gupta S Kugathasan L Ha A Ochoa A Balter M . Cardiac sarcoidosis: a clinical overview. Curr Prob Cardiol. (2021) 46:100936. 10.1016/j.cpcardiol.2021.100936 34400001

[46] Nunes H Humbert M Capron F Brauner M Sitbon O Battesti JP . Pulmonary hypertension associated with sarcoidosis: mechanisms, haemodynamics and prognosis. Thorax. (2006) 61:68–74. 10.1136/thx.2005.042838 16227329 PMC2080703

[47] Takemura T Matsui Y Saiki S Mikami R. Pulmonary vascular involvement in sarcoidosis: a report of 40 autopsy cases. Human Pathol. (1992) 23:1216–23. 10.1016/0046-8177(92)90288-E 1427751

[48] Weatherald J Dorfmüller P Perros F Ghigna MR Girerd B Humbert M . Pulmonary capillary haemangiomatosis: a distinct entity? Eur Res Rev. (2020) 29:190168. 10.1183/16000617.0168-2019 32461209 PMC9488541

[49] Ota H Sugino K Uekusa T Takemura T Homma S. An autopsy case of refractory pulmonary hypertension with sarcoidosis. Res Inv. (2016) 54:490–3. 10.1016/j.resinv.2016.05.005 27886864

[50] Hoffstein V Ranganathan N Mullen JB. Sarcoidosis simulating pulmonary veno-occlusive disease. Am Rev Res Dis. (1986) 134:809–11. 3767134 10.1164/arrd.1986.134.4.809

[51] Ungprasert P Crowson CS Matteson EL. Association of sarcoidosis with increased risk of VTE: a population-based study, 1976 to 2013. Chest. (2017) 151:425–30. 10.1016/j.chest.2016.09.009 27687848 PMC5310113

[52] Goljan-Geremek A Geremek M Puscinska E Sliwinski P. Venous thromboembolism and sarcoidosis: co-incidence or coexistence? Cent Eur J Immunol. (2015) 40:477–80. 10.5114/ceji.2015.56972 26862313 PMC4737745

[53] Swigris JJ Olson AL Huie TJ Fernandez-Perez ER Solomon JJ Sprunger D . Increased risk of pulmonary embolism among US decedents with sarcoidosis from 1988 to 2007. Chest. (2011) 140:1261–6. 10.1378/chest.11-0324 21565969 PMC3205849

[54] Duong H Bonham CA. Sarcoidosis-associated pulmonary hypertension: pathophysiology, diagnosis, and treatment. Clin Pulm Med. (2018) 25:52–60. 10.1097/CPM.0000000000000252 30294198 PMC6168942

[55] Ozyilmaz E Akilli R Berk I Deniz A Ozturk OG Baydar O . The frequency of diastolic dysfunction in patients with sarcoidosis and it's relationship with HLA DRB1^*^ alleles. Sarcoid Vasc Diffuse Lung Dis. (2019) 36:285–93. 10.36141/svdld.v36i4.8606 32476964 PMC7247094

[56] Lal C Medarov BI Judson MA. Interrelationship between sleep-disordered breathing and sarcoidosis. Chest. (2015) 148:1105–14. 10.1378/chest.15-0584 25996391

[57] Simonneau G Montani D Celermajer DS Denton CP Gatzoulis MA Krowka M . Haemodynamic definitions and updated clinical classification of pulmonary hypertension. Eur Respir J. (2019) 53:1801913. 10.1183/13993003.01913-2018 30545968 PMC6351336

[58] Shorr AF Davies DB Nathan SD. Predicting mortality in patients with sarcoidosis awaiting lung transplantation. Chest. (2003) 124:922–8. 10.1016/S0012-3692(15)37649-2 12970018

[59] Neville E Walker AN James DG. Prognostic factors predicting the outcome of sarcoidosis: an analysis of 818 patients. Quart J Med. (1983) 52:525–33. 6657915

[60] Mathijssen H Huitema MP Bakker ALM Mager JJ Snijder RJ Grutters JC . Safety of macitentan in sarcoidosis-associated pulmonary hypertension: a case-series. Sarcoid Vasc Diffuse Lung Dis. (2020) 37:74–8. 10.1093/ehjci/ehaa946.2231 33093771 PMC7569544

[61] Keir GJ Walsh SL Gatzoulis MA Marino PS Dimopoulos K Alonso R . Treatment of sarcoidosis-associated pulmonary hypertension: a single centre retrospective experience using targeted therapies. Sarcoid Vasc Diffuse Lung Dis. (2014) 31:82–90. 25078636

[62] Baughman RP Culver DA Cordova FC Padilla M Gibson KF Lower EE . Bosentan for sarcoidosis-associated pulmonary hypertension: a double-blind placebo controlled randomized trial. Chest. (2014) 145:810–7. 10.1378/chest.13-1766 24177203

[63] Baughman RP Judson MA Lower EE Highland K Kwon S Craft N . Inhaled iloprost for sarcoidosis associated pulmonary hypertension. Sarcoid Vasc Diffuse Lung Dis. (2009) 26:110–20. 20560291

[64] Fisher KA Serlin DM Wilson KC Walter RE Berman JS Farber HW. Sarcoidosis-associated pulmonary hypertension: outcome with long-term epoprostenol treatment. Chest. (2006) 130:1481–8. 10.1378/chest.130.5.1481 17099027

[65] Hendriks S Peetoom K Bakker C van der Flier WM Papma JM Koopmans R . Global prevalence of young-onset dementia: a systematic review and meta-analysis. JAMA Neurol. (2021) 78:1080–90. 10.1001/jamaneurol.2021.2161 34279544 PMC8290331

[66] Wu M Tong X Liu S Wang D Wang L Fan H. Prevalence of methicillin-resistant staphylococcus aureus in healthy Chinese population: a system review and meta-analysis. PLoS ONE. (2019) 14:e0223599. 10.1371/journal.pone.0223599 31647842 PMC6812772

