# Supplementary figures and images for: Prevalence of Sarcoidosis-Associated Pulmonary Hypertension: A Systematic Review and Meta-Analysis
Source: Front Cardiovasc Med. 2022 Jan 17;8:809594. doi: 10.3389/fcvm.2021.809594 (PMC8801498; doi:10.3389/fcvm.2021.809594)

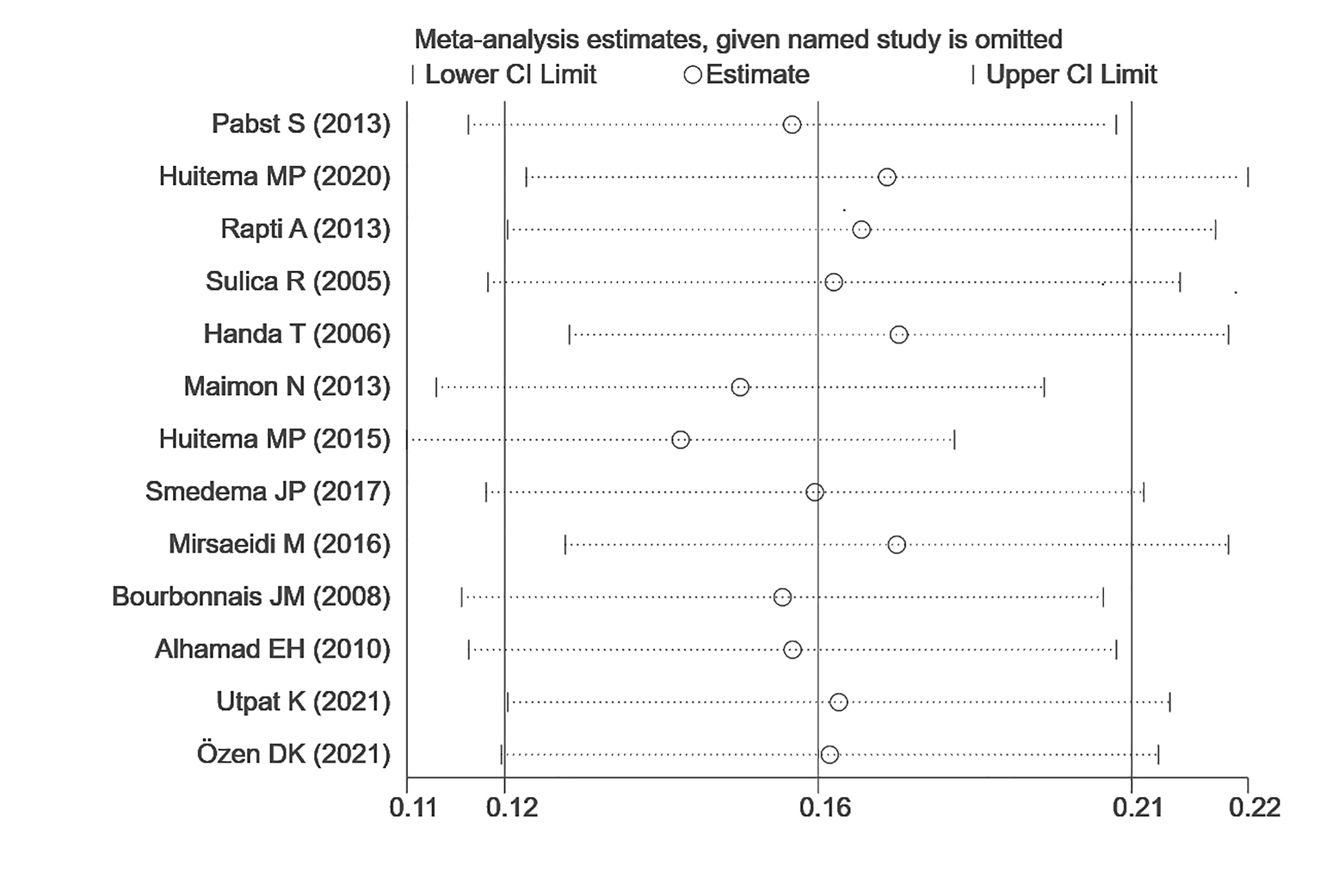

Supplement: Supplementary Figure 1 — Results of the sensitivity analysis of the prevalence of SAPH by TTE. SAPH, sarcoidosis-associated pulmonary hypertension; TTE, transthoracic echocardiography. [file Data_Sheet_1.ZIP › Supplementary Figures/Supplementary Figure 1.tif]

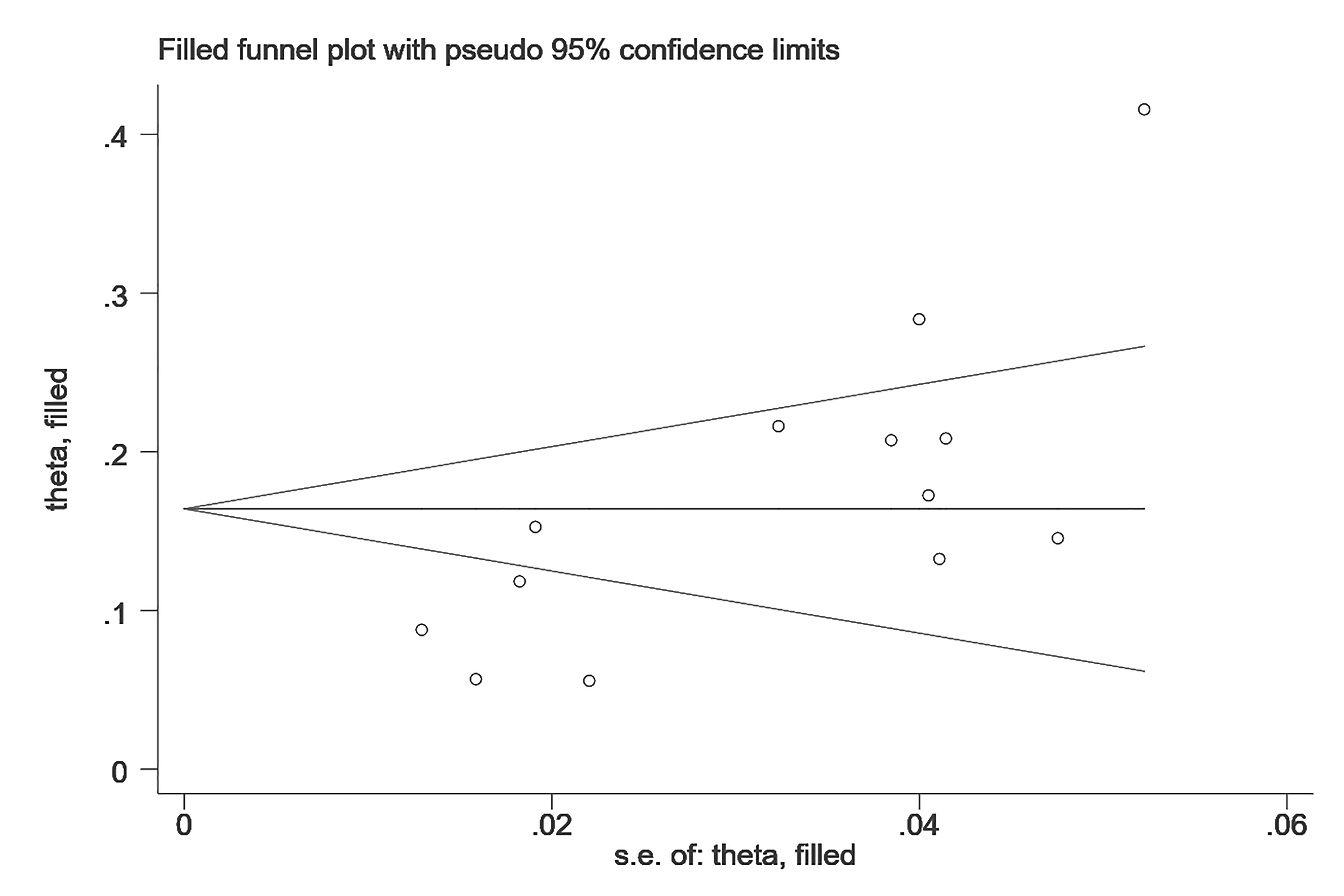

Supplement: Supplementary Figure 1 — Results of the sensitivity analysis of the prevalence of SAPH by TTE. SAPH, sarcoidosis-associated pulmonary hypertension; TTE, transthoracic echocardiography. [file Data_Sheet_1.ZIP › Supplementary Figures/Supplementary Figure 2.tif]

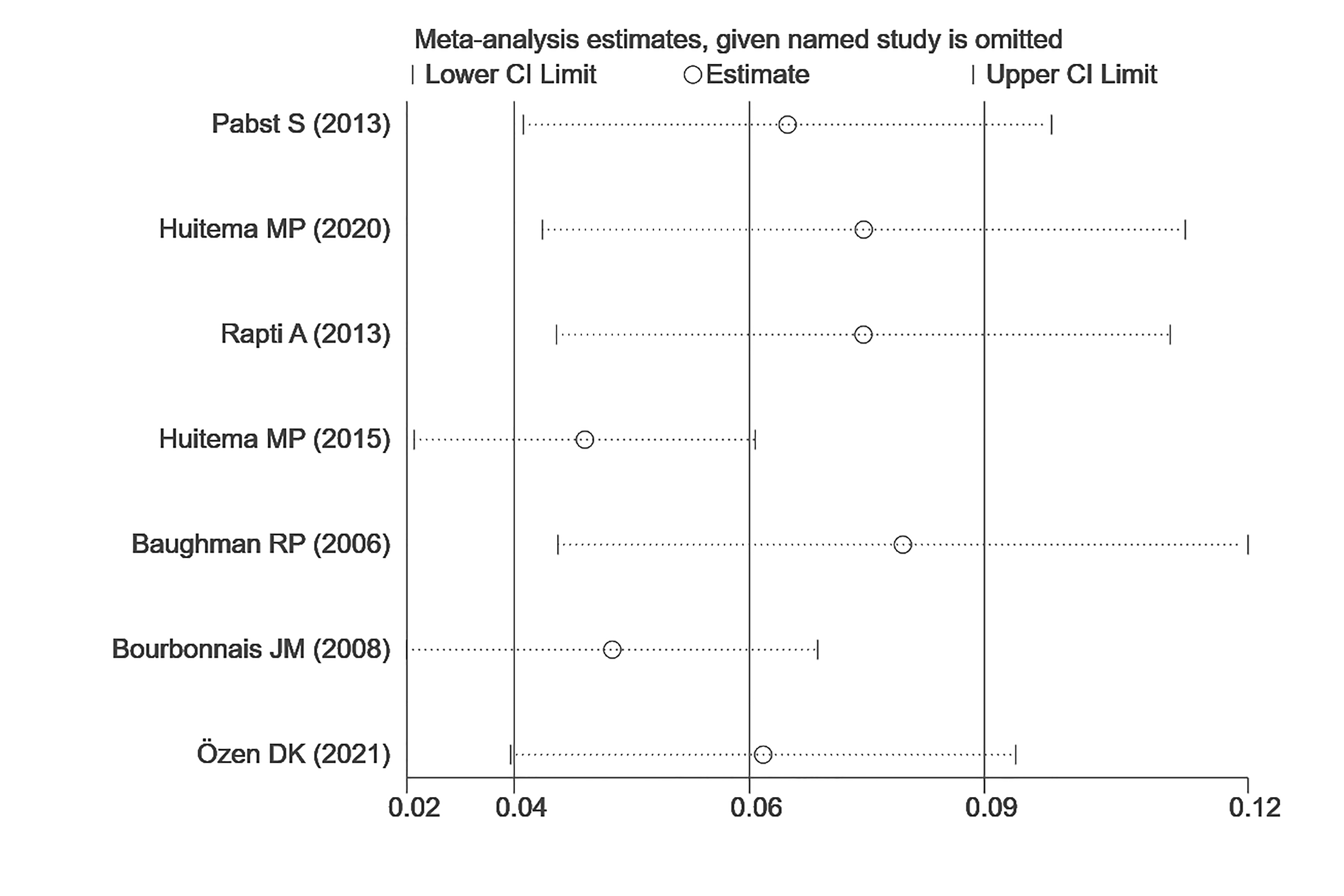

Supplement: Supplementary Figure 1 — Results of the sensitivity analysis of the prevalence of SAPH by TTE. SAPH, sarcoidosis-associated pulmonary hypertension; TTE, transthoracic echocardiography. [file Data_Sheet_1.ZIP › Supplementary Figures/Supplementary Figure 3.tif]

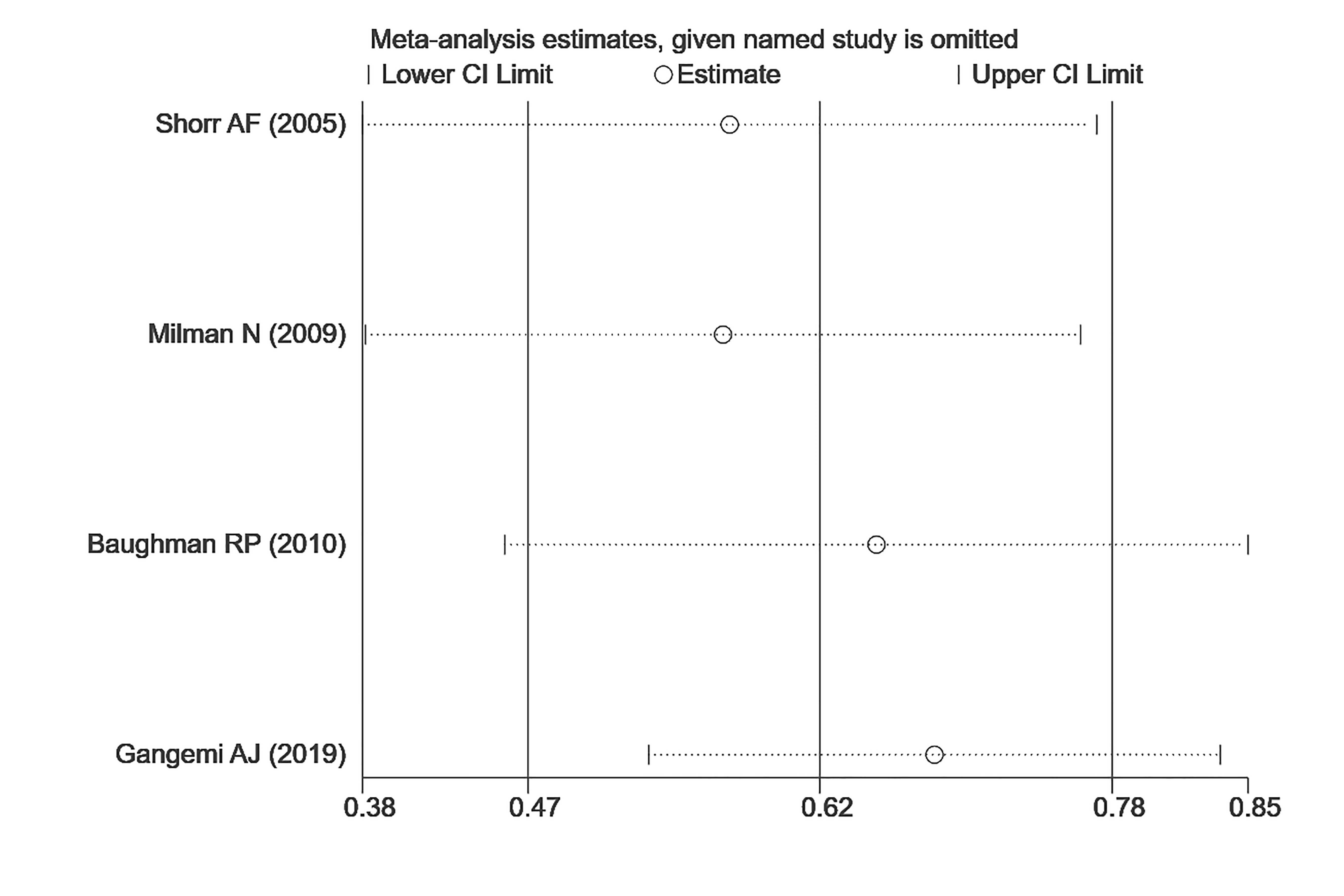

Supplement: Supplementary Figure 1 — Results of the sensitivity analysis of the prevalence of SAPH by TTE. SAPH, sarcoidosis-associated pulmonary hypertension; TTE, transthoracic echocardiography. [file Data_Sheet_1.ZIP › Supplementary Figures/Supplementary Figure 4.tif]

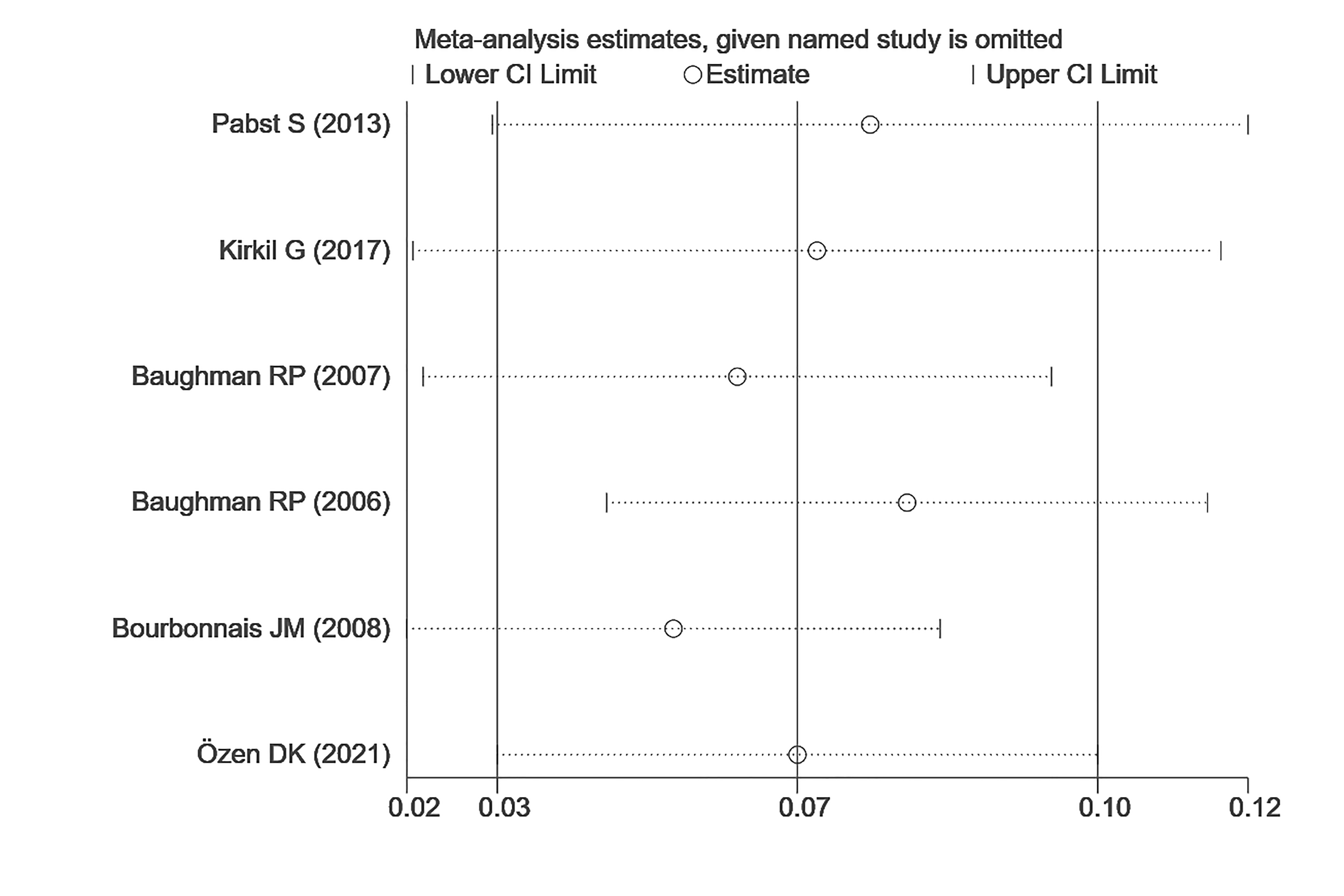

Supplement: Supplementary Figure 1 — Results of the sensitivity analysis of the prevalence of SAPH by TTE. SAPH, sarcoidosis-associated pulmonary hypertension; TTE, transthoracic echocardiography. [file Data_Sheet_1.ZIP › Supplementary Figures/Supplementary Figure 5.tif]

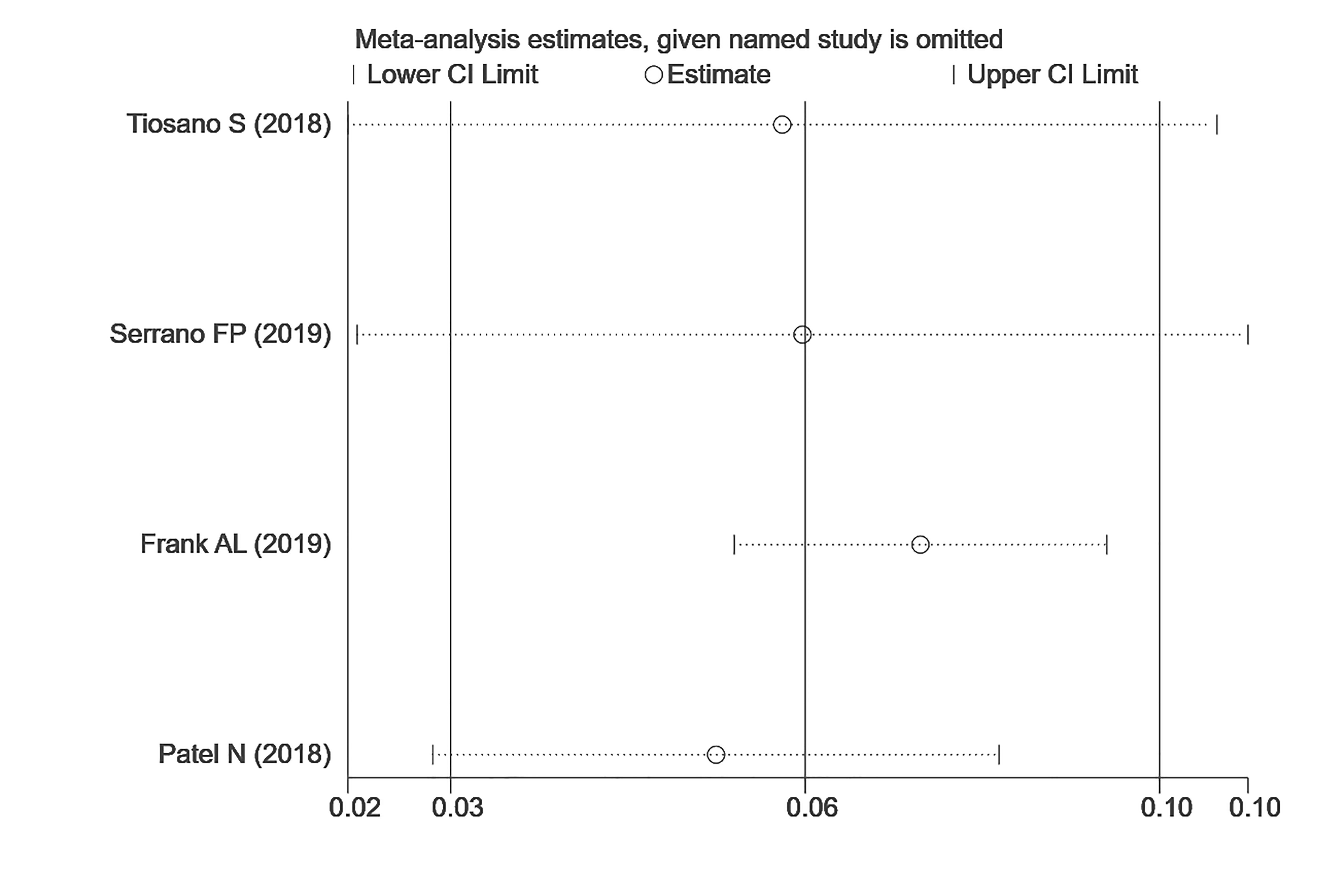

Supplement: Supplementary Figure 1 — Results of the sensitivity analysis of the prevalence of SAPH by TTE. SAPH, sarcoidosis-associated pulmonary hypertension; TTE, transthoracic echocardiography. [file Data_Sheet_1.ZIP › Supplementary Figures/Supplementary Figure 6.tif]

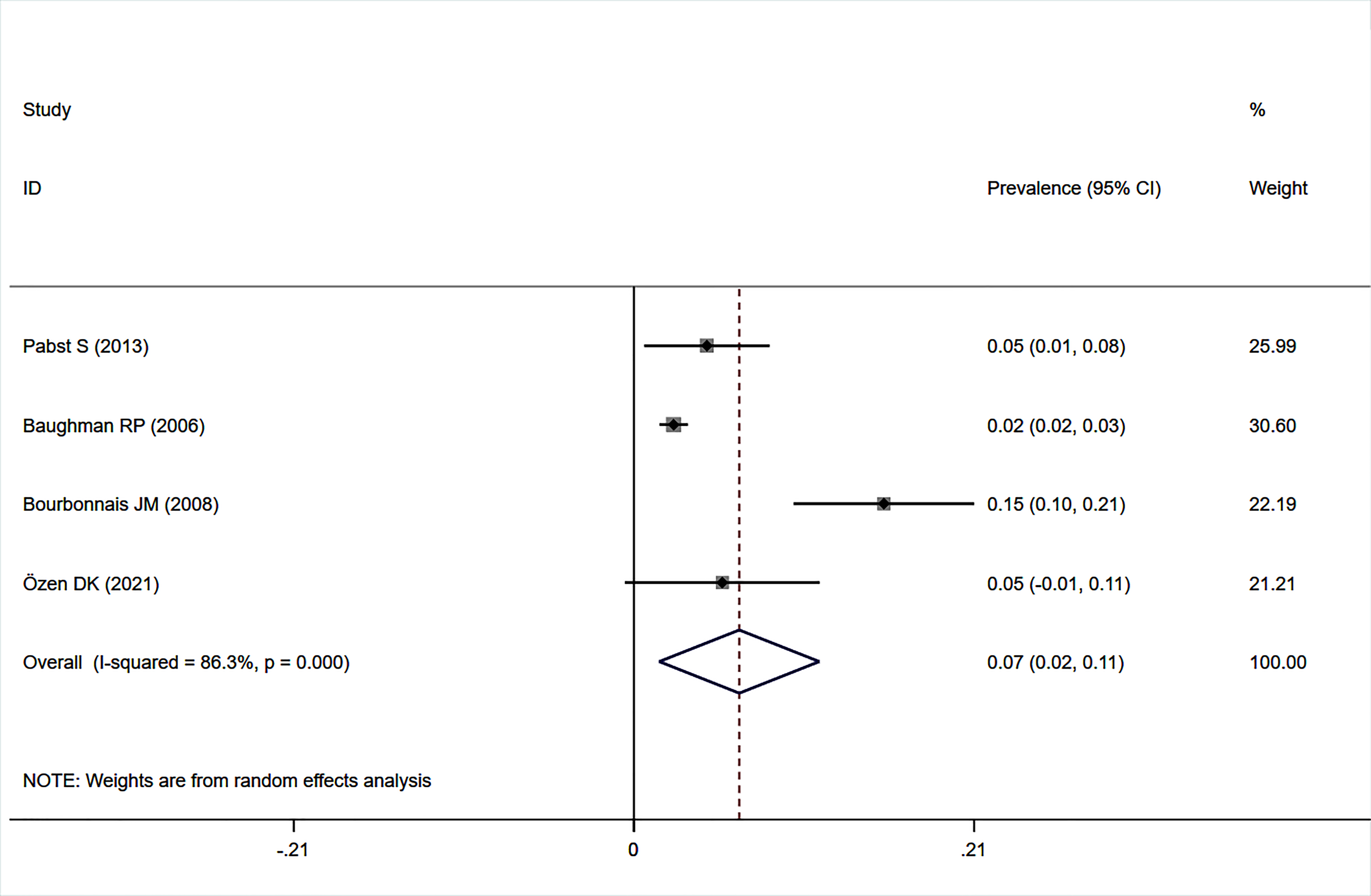

Supplement: Supplementary Figure 1 — Results of the sensitivity analysis of the prevalence of SAPH by TTE. SAPH, sarcoidosis-associated pulmonary hypertension; TTE, transthoracic echocardiography. [file Data_Sheet_1.ZIP › Supplementary Figures/Supplementary Figure 7.tif]

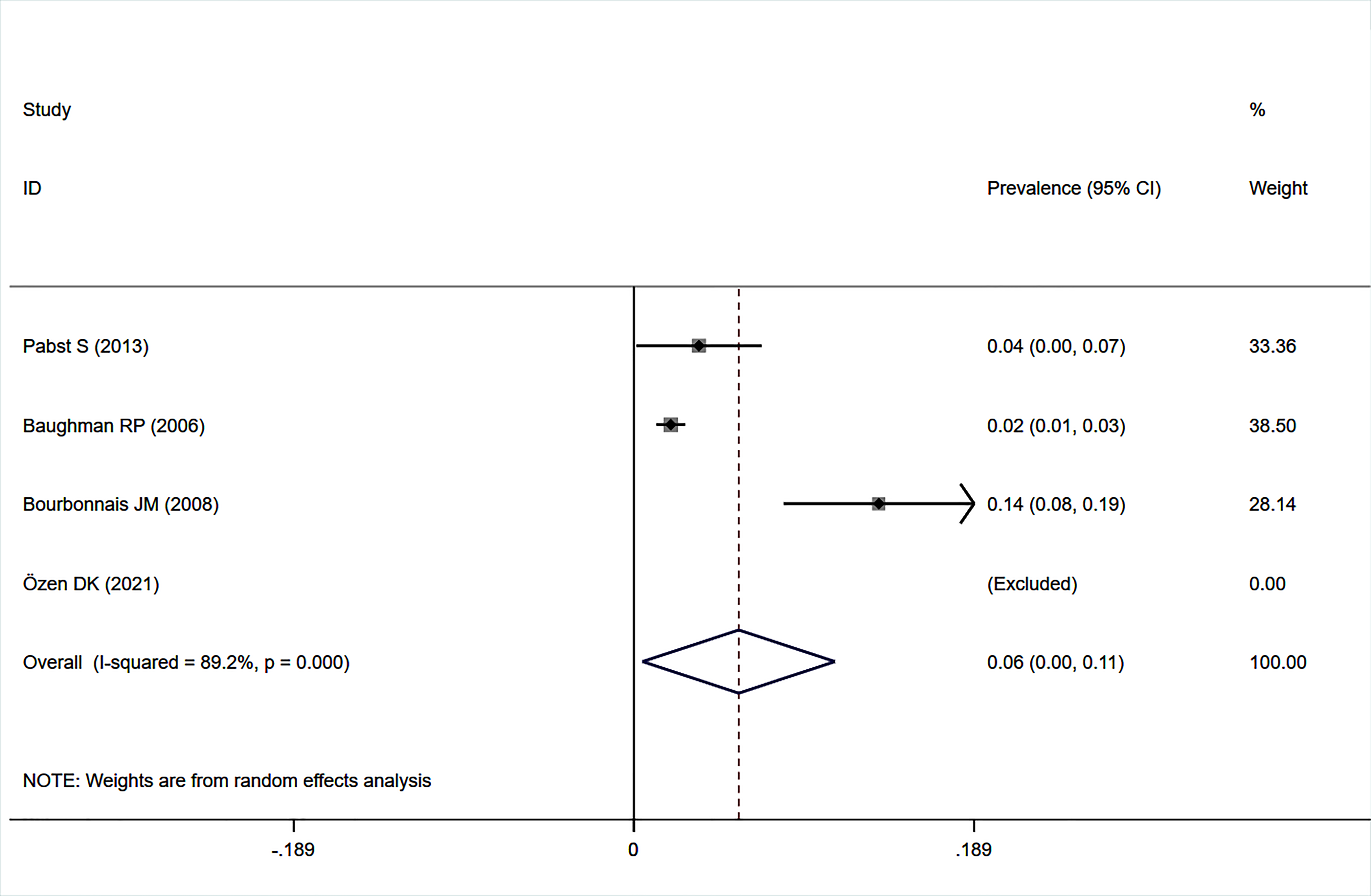

Supplement: Supplementary Figure 1 — Results of the sensitivity analysis of the prevalence of SAPH by TTE. SAPH, sarcoidosis-associated pulmonary hypertension; TTE, transthoracic echocardiography. [file Data_Sheet_1.ZIP › Supplementary Figures/Supplementary Figure 8.tif]
