# Supplementary material for: Prevalence of Sarcoidosis-Associated Pulmonary Hypertension: A Systematic Review and Meta-Analysis
Source: Front Cardiovasc Med. 2022 Jan 17;8:809594. doi: 10.3389/fcvm.2021.809594 (PMC8801498; doi:10.3389/fcvm.2021.809594)
Supplement: Supplementary Table 1 — MOOSE checklist (15). [file Data_Sheet_2.zip › Supplementary Tables 2/Supplementary Table 1. MOOSE checklist..docx]

**Supplementary Table 1. MOOSE checklist [1].**

| **Item No** | **Recommendation** | **Reported**  **(√or ×)** |
| --- | --- | --- |
| Reporting of background should include | | |
| 1 | Problem definition | **√** |
| 2 | Hypothesis statement | N/A |
| 3 | Description of study outcome(s) | **√** |
| 4 | Type of exposure or intervention used | **√** |
| 5 | Type of study designs used | **√** |
| 6 | Study population | **√** |
| Reporting of search strategy should include | | |
| 7 | Qualifications of searchers (eg, librarians and investigators) | **√** |
| 8 | Search strategy, including time period included in the synthesis and key words | **√** |
| 9 | Effort to include all available studies, including contact with authors | **√** |
| 10 | Databases and registries searched | **√** |
| 11 | Search software used, name and version, including special features used (eg, explosion) | **√** |
| 12 | Use of hand searching (eg, reference lists of obtained articles) | **√** |
| 13 | List of citations located and those excluded, including justification | **√** |
| 14 | Method of addressing articles published in languages other than English | **√** |
| 15 | Method of handling abstracts and unpublished studies | **√** |
| 16 | Description of any contact with authors | **√** |
| Reporting of methods should include | | |
| 17 | Description of relevance or appropriateness of studies assembled for assessing the hypothesis to be tested | **√** |
| 18 | Rationale for the selection and coding of data (eg, sound clinical principles or convenience) | **√** |
| 19 | Documentation of how data were classified and coded (eg, multiple raters, blinding and interrater reliability) | **√** |
| 20 | Assessment of confounding (eg, comparability of cases and controls in studies where appropriate) | N/A |
| 21 | Assessment of study quality, including blinding of quality assessors, stratification or regression on possible predictors of study results | **√** |
| 22 | Assessment of heterogeneity | **√** |
| 23 | Description of statistical methods (eg, complete description of fixed or random effects models, justification of whether the chosen models account for predictors of study results, dose-response models, or cumulative meta-analysis) in sufficient detail to be replicated | **√** |
| 24 | Provision of appropriate tables and graphics | **√** |
| Reporting of results should include | | |
| 25 | Graphic summarizing individual study estimates and overall estimate | **√** |
| 26 | Table giving descriptive information for each study included | **√** |
| 27 | Results of sensitivity testing (eg, subgroup analysis) | **√** |
| 28 | Indication of statistical uncertainty of findings | **√** |
| Reporting of discussion should include | | |
| 29 | Quantitative assessment of bias (eg, publication bias) | √ |
| 30 | Justification for exclusion (eg, exclusion of non-English language citations) | √ |
| 31 | Assessment of quality of included studies | √ |
| Reporting of conclusions should include | | |
| 32 | Consideration of alternative explanations for observed results | **√** |
| 33 | Generalization of the conclusions (ie, appropriate for the data presented and within the domain of the literature review) | **√** |
| 34 | Guidelines for future research | **√** |
| 35 | Disclosure of funding source | **√** |

N/A: Not applicable

Reference:

1. Stroup DF, Berlin JA, Morton SC, Olkin I, Williamson GD, Rennie D, Moher D, Becker BJ, Sipe TA, Thacker SB: **Meta-analysis of observational studies in epidemiology: a proposal for reporting. Meta-analysis Of Observational Studies in Epidemiology (MOOSE) group.** *Jama* 2000, **283:**2008-2012.
