# Supplementary material for: Prevalence of Sarcoidosis-Associated Pulmonary Hypertension: A Systematic Review and Meta-Analysis
Source: Front Cardiovasc Med. 2022 Jan 17;8:809594. doi: 10.3389/fcvm.2021.809594 (PMC8801498; doi:10.3389/fcvm.2021.809594)
Supplement: Supplementary Table 1 — MOOSE checklist (15). [file Data_Sheet_2.zip › Supplementary Tables 2/Supplementary Table 3..docx]

**Supplementary Table 3. Diagnostic criteria of pulmonary hypertension in included studies.**

| Study | Year | Time period | SAPH was suspected by TTE | SAPH was diagnosed by RHC |  |
| --- | --- | --- | --- | --- | --- |
| Pabst S | 2013 | January 2010 to October 2010 | Elevated estimation of PAPsyst ≥30 mmHg (+ estimated central venous pressure) with dyspnea or a PAPsyst ≥50 mmHg (+ estimated central venous pressure) without dyspnea | A mPAP ≥ 25 mmHg regardless of the PCWP |  |
| Shorr AF | 2005 | January 1995 to December 2002 | NR | A mPAP of＞25 mmHg |  |
| Huitema MP | 2020 | August 2015 to November 2018 | Intermediate and high PH probability according to the 2015 ESC/ERS PH guideline for PH | A mPAP of ≥ 25 mm Hg |  |
| Rapti A | 2013 | October 2002 to June 2010 | Pulmonary artery systolic pressure ≥40 mmHg by | A mPAP ≥25 mmHg at rest |  |
| Milman N | 2009 | January 1992 to December 2006 | NR | A mPAP >25 mmHg |  |
| Sulica R | 2005 | January 1997 to December 2001 | Estimated RVSP of at least 40 mm Hg, which corresponds to a peak tricuspid regurgitant velocity of 3.0 to 3.5 m/s | NR |  |
| Handa T | 2006 | August 2004 to April 2005 | Estimated PAPsyst > 40 mm Hg | NR |  |
| Maimon N | 2013 | October 2009 to May 2011 | Estimated RVSP > 40 mmHg | NR |  |
| Baughman RP | 2010 | NR | NR | A mPAP of ≥ 25 mm Hg |  |
| Kirkil G | 2017 | the year 2002 to 2008 | NR | A mPAP of＞25 mm Hg |  |
| Huitema MP | 2015 | November 2007 to May 2014 | Estimated RVSP >36 mmHg, or presence of secondary signs with normal/absent RVSP signal | A mPAP of ≥ 25 mm Hg |  |
| Smedema JP | 2017 | July 2001 to March 2014 | Peak systolic right ventricular pressures＞35 mmHg | NR |  |
| Baughman RP | 2007 | June 2005 to July 2005 | NR | A mPAP of＞25 mm Hg |  |
| Nardi A | 2011 | April 1986 to May 2006 | Estimated PAPsyst of ＞40 mmHg | NR |  |
| Mirsaeidi M | 2016 | January 2010 to January 2015 | Pulmonary artery systolic pressure ≥ 50mmHg | NR |  |
| Baughman RP | 2006 | NR | NR | A mPAP of > 25 mm Hg |  |
| Bourbonnais JM | 2008 | NR | A RVSP＞40 mmHg in the absence of significant left heart dysfunction | NR |  |
| Alhamad EH | 2010 | January 2001 to December 2008 | An estimated RVSP of >40 mm Hg in the absence of left ventricular dysfunction, ischemic heart disease or valvular heart disease | NR |  |
| Utpat K | 2021 | NR | An estimated moderate and severe PH by Two-dimensional echocardiography | NR |  |
| Özen DK | 2021 | NR | Intermediate-high risk of PH according to the 2015 ESC/ERS PH guideline for PH | A mPAP of >20 mmHg |  |
| Tiosano S | 2018 | the year 2000 to 2016 | NR | A mPAP ≥ 25 mmHg at rest |  |
| Gangemi AJ* | 2019 | March 2012 to February 2019 | PH was determined by a secondary listing diagnosis. | |  |
| Serrano FP* | 2019 | January 2014 to December 2015 | PH was determined by ICD-9-CM codes (416.0 and 416.8). | |  |
| Frank AL* | 2019 | the year 2009 to 2014 | PH was determined by ICD-10-CM codes (I27.0, I27.8, and I27.9). | |  |
| Patel N* | 2018 | the year 2005 to 2014. | PH was determined by ICD-9-CM Codes (416.0, 416.8, and 416.9). | |  |

Note: NR, not reported; PAPsyst, systolic pulmonary arterial pressure; PCWP, pulmonary capillary wedge pressure; mPAP, mean pulmonary artery pressure; RVSP, right ventricular systolic pressure; PH, pulmonary hypertension.

*Pulmonary hypertension was diagnosed in other ways.
