# Supplementary material for: Prevalence of Sarcoidosis-Associated Pulmonary Hypertension: A Systematic Review and Meta-Analysis
Source: Front Cardiovasc Med. 2022 Jan 17;8:809594. doi: 10.3389/fcvm.2021.809594 (PMC8801498; doi:10.3389/fcvm.2021.809594)
Supplement: Supplementary Table 1 — MOOSE checklist (15). [file Data_Sheet_2.zip › Supplementary Tables 2/Supplementary Table 5..docx]

**Supplementary Table 5. Quality assessment of included studies.**

| Study | (Selection bias) | | | nonresponse bias | measurement bias | | | | | Bias related to analysis | Total scores |
| --- | --- | --- | --- | --- | --- | --- | --- | --- | --- | --- | --- |
|  | Representativeness | Sampling frame | Random selection |  | Informant | Case definition | Validity and reliability of measures | Mode of data collection | Prevalence period |  |  |
| Pabst S (2013) | High | Low | Low | Low | Low | Low | Low | Low | Low | Low | 1 |
| Shorr AF (2005) | Low | Low | Low | Low | High | Low | Low | Low | Low | Low | 1 |
| Huitema MP (2020) | High | Low | Low | Low | Low | Low | Low | Low | Low | Low | 1 |
| Rapti A (2013) | High | Low | Low | Low | Low | Low | Low | Low | Low | Low | 1 |
| Milman N (2009) | High | Low | Low | Low | Low | Low | Low | Low | Low | Low | 1 |
| Sulica R (2005) | High | Low | Low | Low | Low | Low | Low | Low | Low | Low | 1 |
| Handa T (2006) | High | Low | Low | Low | Low | Low | Low | Low | Low | Low | 1 |
| Maimon N (2013) | High | Low | Low | Low | Low | Low | Low | Low | Low | Low | 1 |
| Baughman RP (2010) | High | Low | Low | Low | Low | Low | Low | Low | Low | Low | 1 |
| Gangemi AJ (2019) | High | Low | Low | Low | Low | Low | Low | Low | Low | Low | 1 |
| Kirkil G (2017) | High | Low | Low | Low | Low | Low | Low | Low | Low | Low | 1 |
| Huitema MP (2015) | High | Low | Low | Low | Low | High | Low | Low | Low | Low | 2 |
| Smedema JP (2017) | High | Low | Low | Low | Low | Low | Low | Low | Low | Low | 1 |
| Baughman RP (2007) | High | Low | Low | Low | Low | Low | Low | Low | Low | Low | 1 |
| Nardi A (2011) | High | Low | Low | Low | Low | Low | Low | Low | Low | Low | 1 |
| Mirsaeidi M (2016) | High | Low | Low | Low | Low | Low | Low | Low | Low | Low | 1 |
| Baughman RP (2006) | High | Low | Low | Low | Low | Low | Low | Low | Low | Low | 1 |
| Bourbonnais JM (2008) | High | Low | Low | Low | Low | Low | Low | Low | Low | Low | 1 |
| Alhamad EH (2010) | High | Low | Low | Low | Low | Low | Low | Low | Low | Low | 1 |
| Utpat K (2021) | High | Low | Low | Low | Low | Low | Low | Low | Low | Low | 1 |
| Özen DK (2021) | High | Low | Low | Low | Low | Low | Low | Low | Low | Low | 1 |
| Tiosano S (2018) | Low | Low | Low | Low | High | High | High | Low | Low | Low | 3 |
| Serrano FP (2019) | Low | Low | Low | Low | Low | Low | High | Low | Low | Low | 1 |
| Frank AL (2019) | Low | Low | Low | Low | High | Low | High | Low | Low | Low | 2 |
| Patel N (2018) | Low | Low | Low | Low | Low | Low | High | Low | Low | Low | 1 |
